# Supplementary material for: Lessons learned and recommendations for data coordination in collaborative research: The CSER consortium experience
Source: HGG Adv. 2022 May 20;3(3):100120. doi: 10.1016/j.xhgg.2022.100120 (PMC9190054; doi:10.1016/j.xhgg.2022.100120)
Supplement: Document S1. Figures S1–S10 and Table S3 [file mmc1.pdf]

HGGA, Volume *3*

Supplemental information

Lessons learned and recommendations for data coordination in collaborative research: The CSER consortium experience

Kathleen D. Muenzen, Laura M. Amendola, Tia L. Kauffman, Kathleen F. Mittendorf, Jeannette T. Bensen, Flavia Chen, Richard Green, Bradford C. Powell, Mark Kvale, Frank Angelo, Laura Farnan, Stephanie M. Fullerton, Jill O. Robinson, Tianran Li, Priyanka Murali, James M.J. Lawlor, Jeffrey Ou, Lucia A. Hindorff, Gail P. Jarvik, and David R. Crosslin

Figure S1. CSER projects, site populations and sequencing modalities.

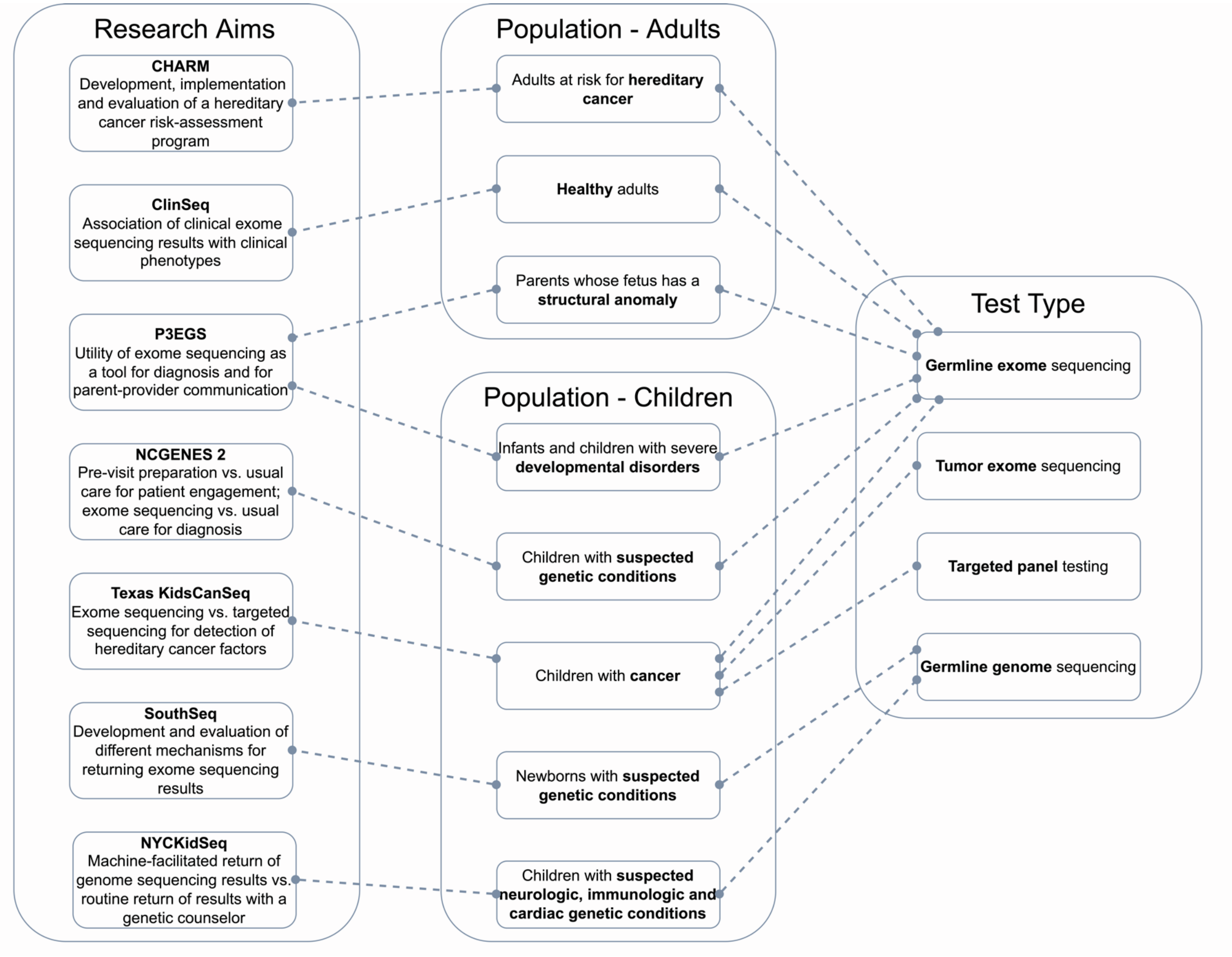

Figure S2. Survey administration timepoints for CSER harmonized survey measures.

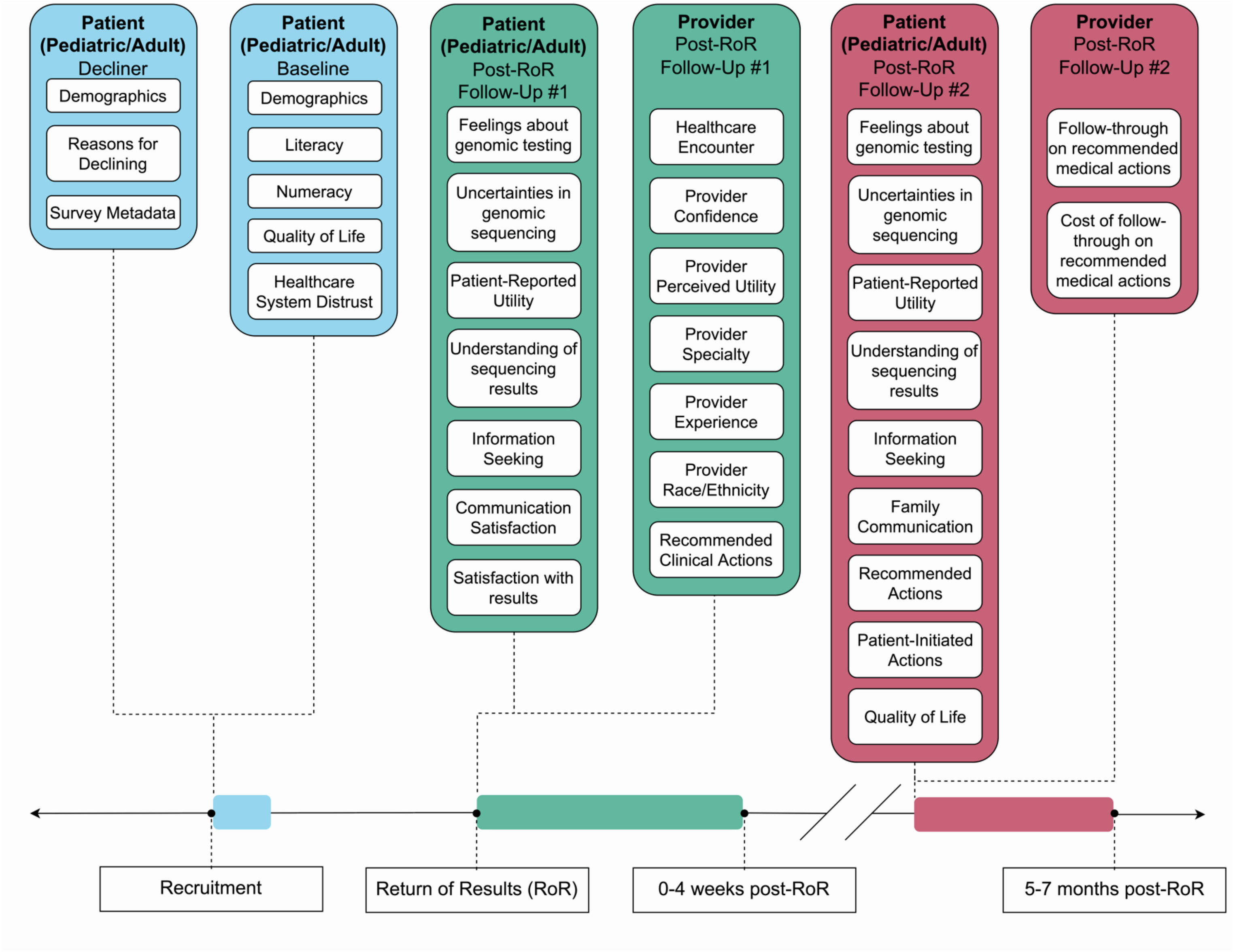

Figure S3. Reporting timepoints for genomic sequencing results, both at the participant level and at the case level.

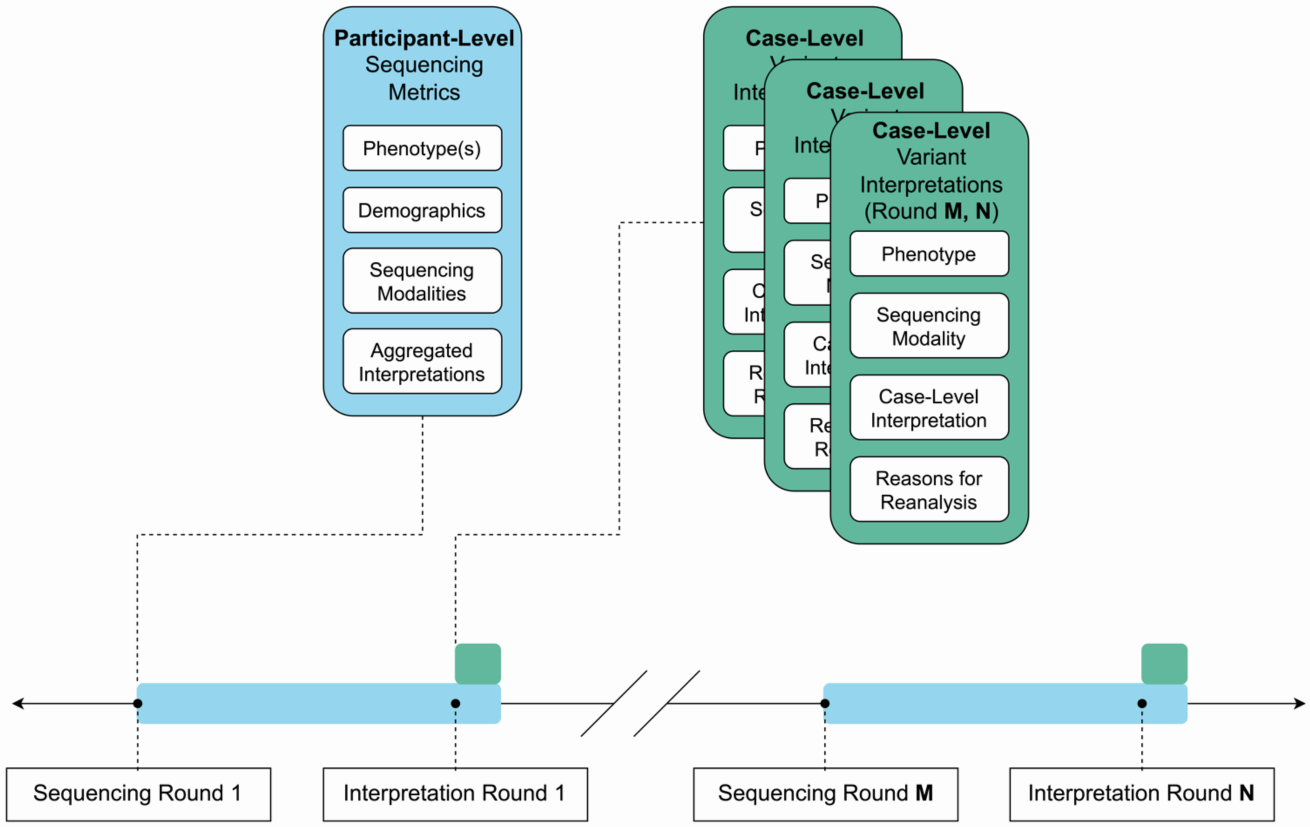

Figure S4. Timeline of the harmonized measure change proposal process and implementation of the post-Return of Results (RoR) to follow-up survey elapsed time variables.

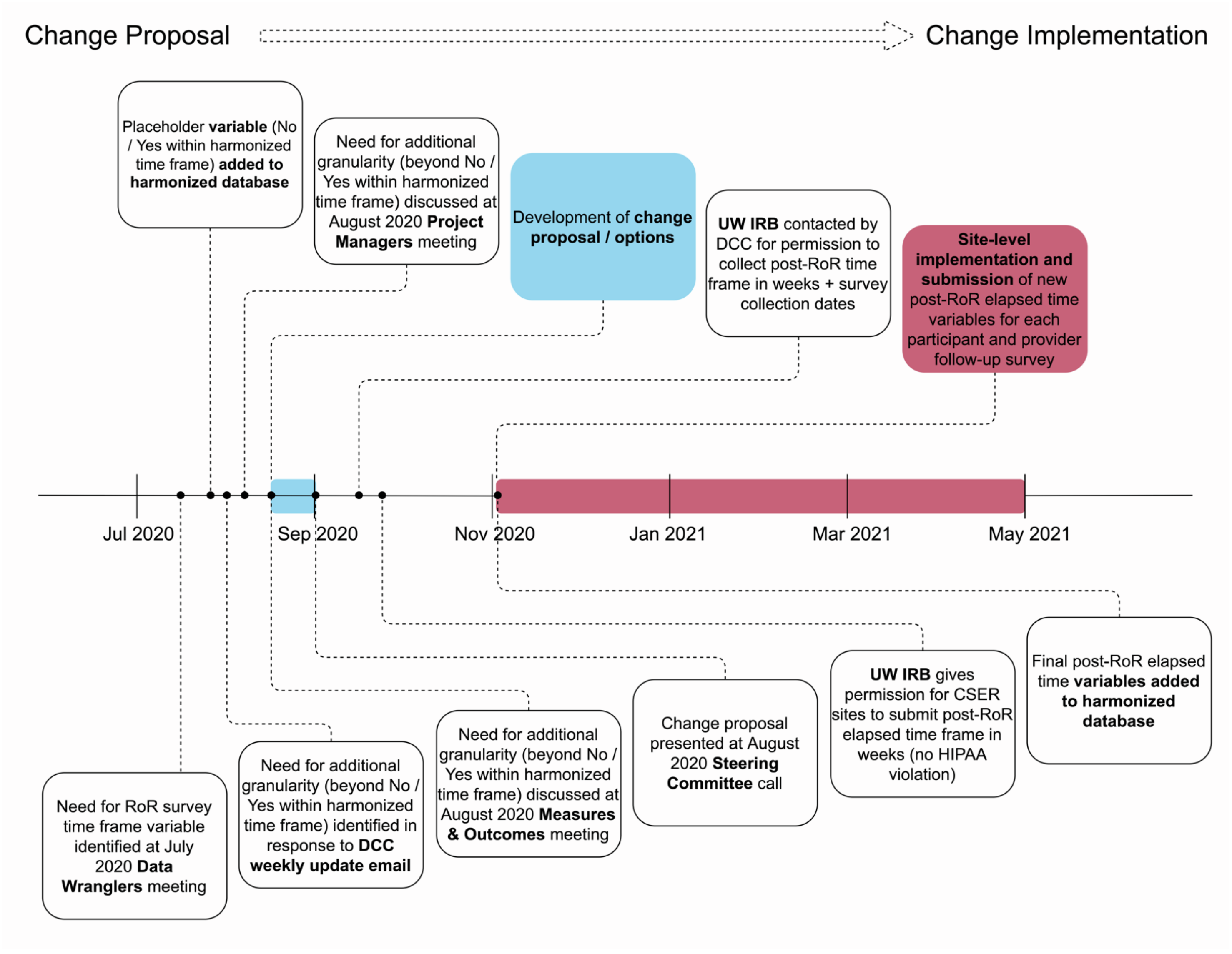

Figure S5. Data upload interface on the CSER Data Hub website.

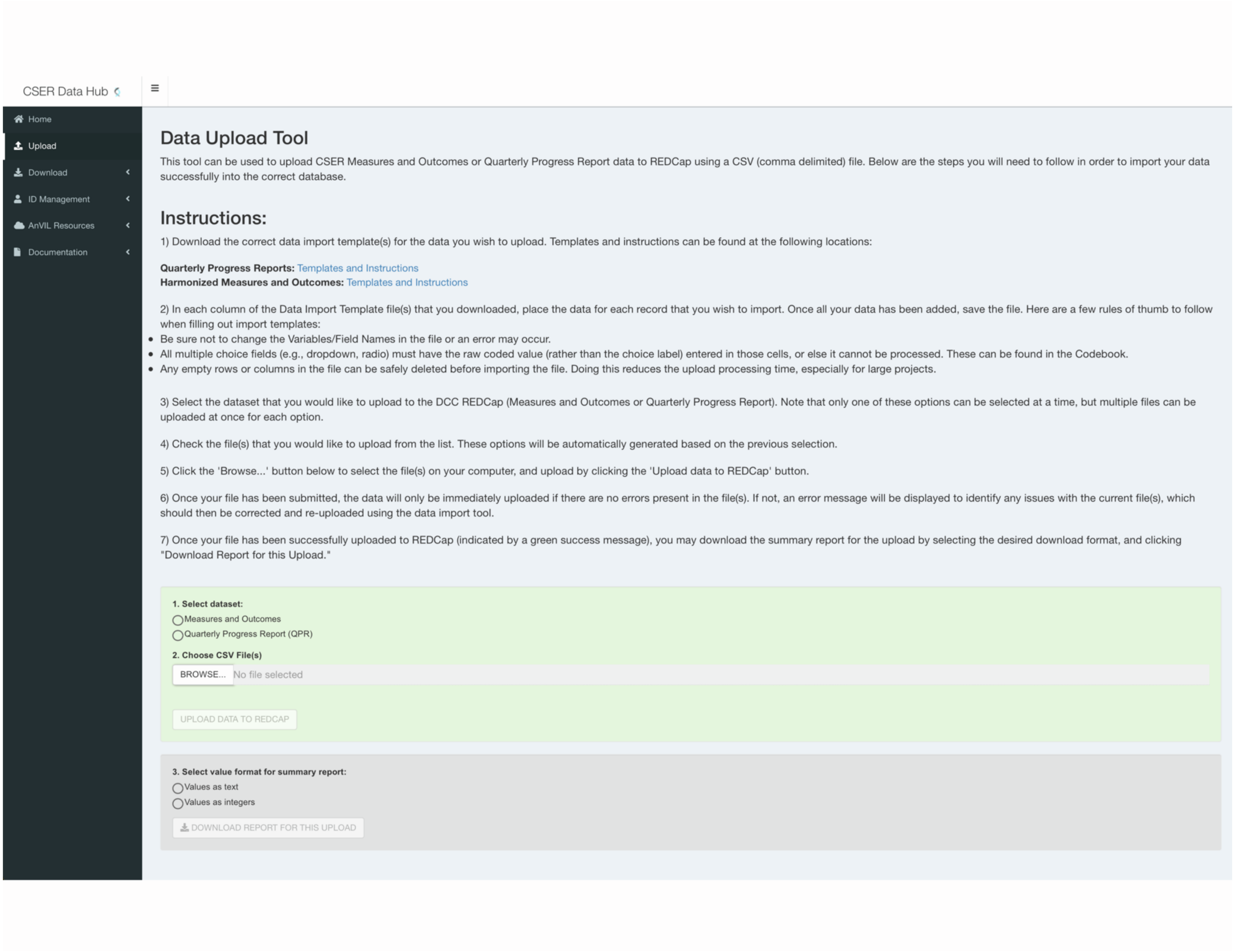

Figure S6. Multi-site harmonized data download interface on the CSER Data Hub website.

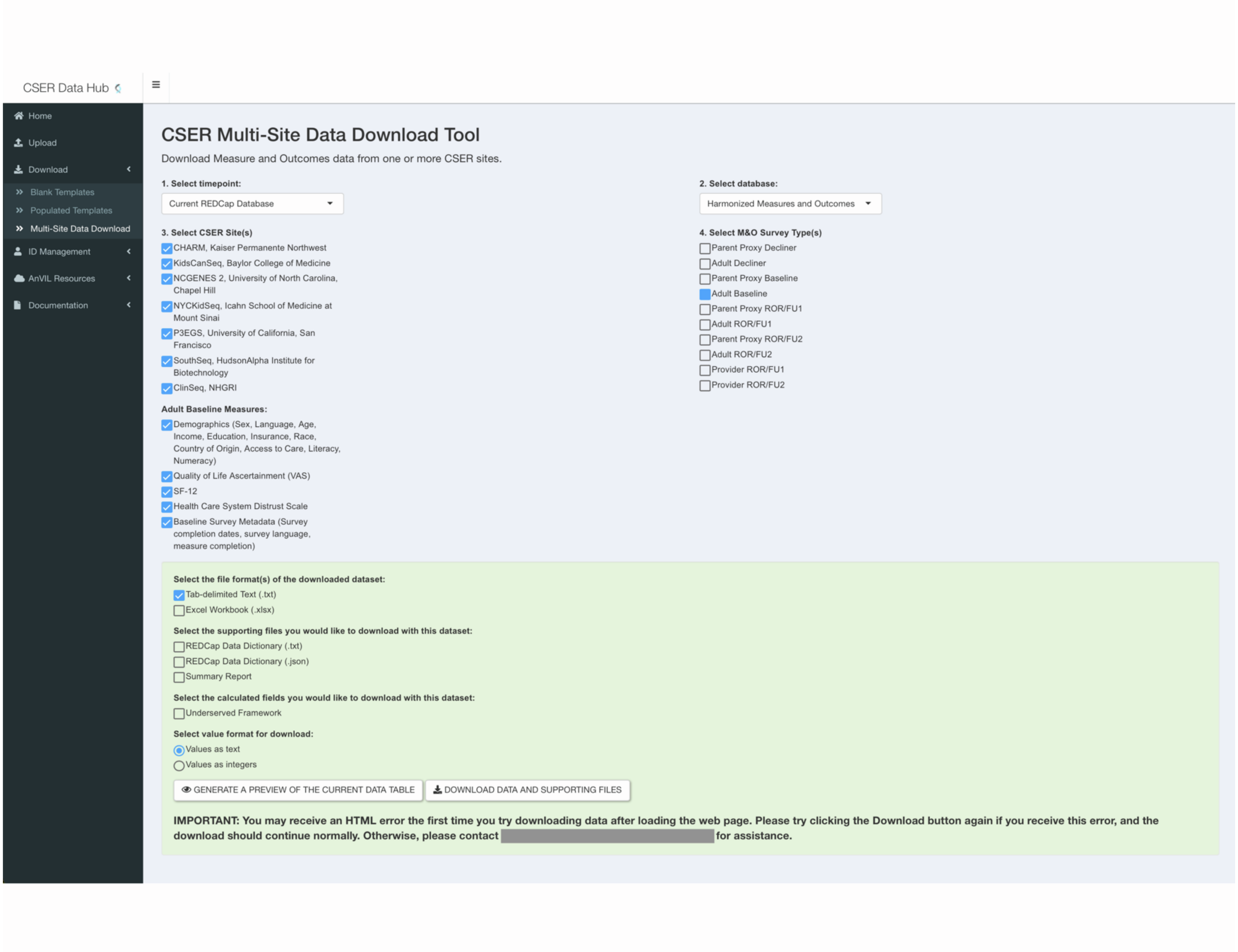

Figure S7. CSER ID management interface on the CSER Data Hub website.

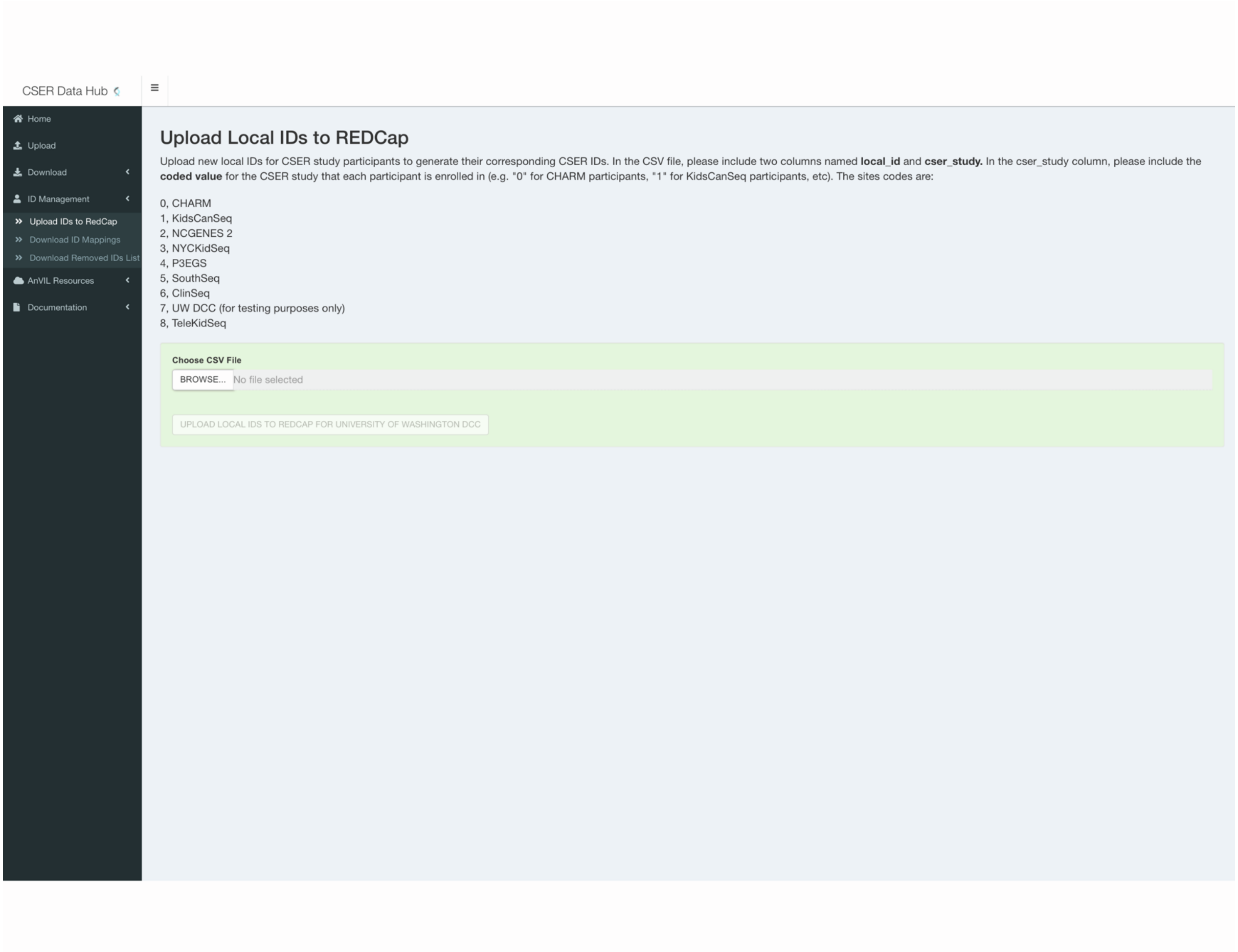

Figure S8. Sequence data upload instructions on the CSER Data Hub website.

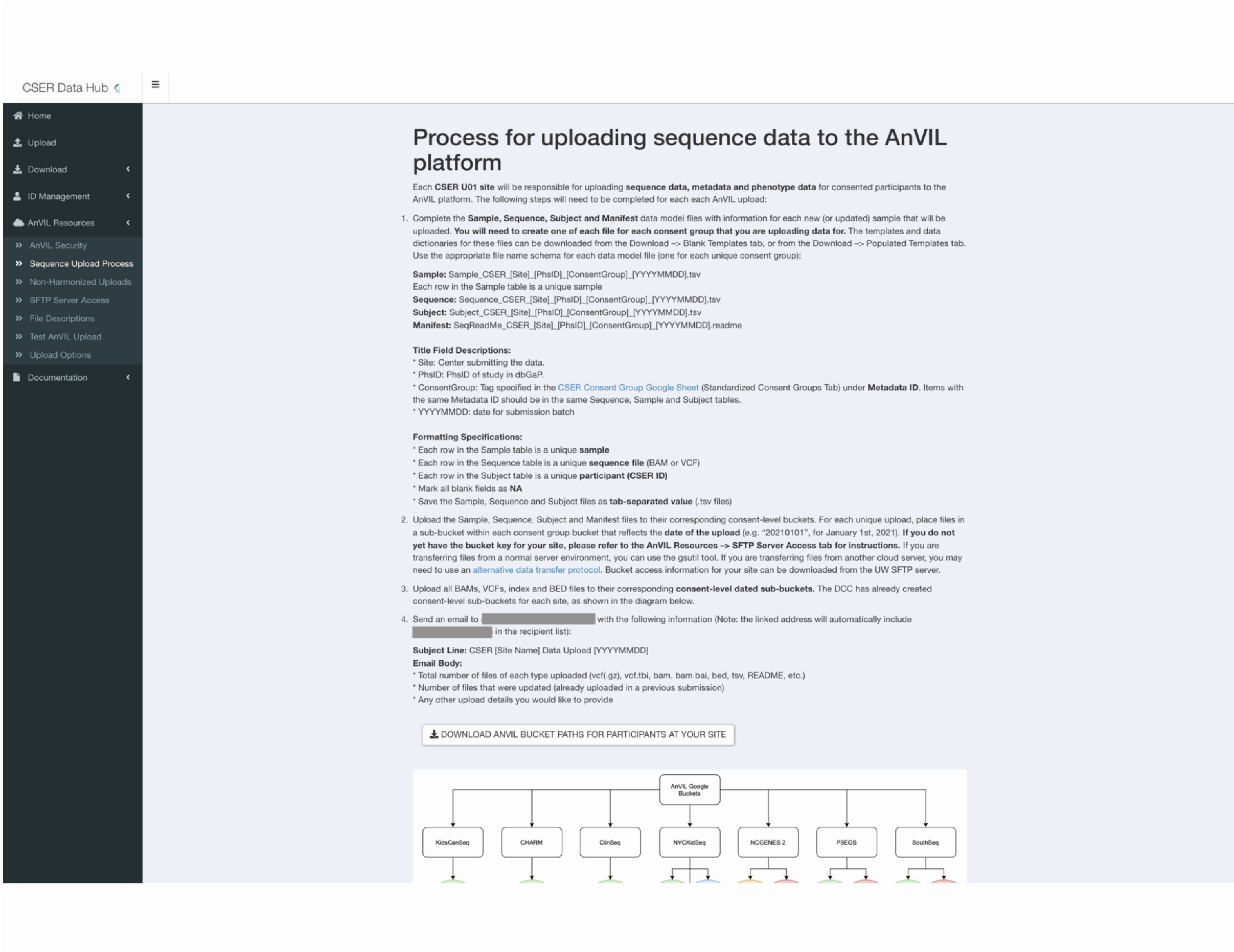
CSER Data Hub

- Home
- Upload
- Download
- ID Management
- AnVIL Resources
  - AnVIL Security
  - Sequence Upload Process
  - Non-Harmonized Uploads
  - SFTP Server Access
  - File Descriptions
  - Test AnVIL Upload
  - Upload Options
- Documentation

Process for uploading sequence data to the AnVIL platform

Each **CSER U01 site** will be responsible for uploading **sequence data, metadata and phenotype data** for consented participants to the AnVIL platform. The following steps will need to be completed for each each AnVIL upload:

1. Complete the **Sample, Sequence, Subject and Manifest** data model files with information for each new (or updated) sample that will be uploaded. **You will need to create one of each file for each consent group that you are uploading data for.** The templates and data dictionaries for these files can be downloaded from the Download –> Blank Templates tab, or from the Download –> Populated Templates tab. Use the appropriate file name schema for each data model file (one for each unique consent group):

   Sample: Sample_CSER_[Site]_[PhsID]_[ConsentGroup]_[YYYYMMDD].tsv
   Each row in the Sample table is a unique sample
   Sequence: Sequence_CSER_[Site]_[PhsID]_[ConsentGroup]_[YYYYMMDD].tsv
   Subject: Subject_CSER_[Site]_[PhsID]_[ConsentGroup]_[YYYYMMDD].tsv
   Manifest: SeqReadMe_CSER_[Site]_[PhsID]_[ConsentGroup]_[YYYYMMDD].readme

   Title Field Descriptions:
   * Site: Center submitting the data.
   * PhsID: PhsID of study in dbGaP.
   * ConsentGroup: Tag specified in the CSER Consent Group Google Sheet (Standardized Consent Groups Tab) under **Metadata ID**. Items with the same Metadata ID should be in the same Sequence, Sample and Subject tables.
   * YYYYMMDD: date for submission batch

   Formatting Specifications:
   * Each row in the Sample table is a unique **sample**
   * Each row in the Sequence table is a unique **sequence file** (BAM or VCF)
   * Each row in the Subject table is a unique **participant (CSER ID)**
   * Mark all blank fields as **NA**
   * Save the Sample, Sequence and Subject files as **tab-separated value** (.tsv files)

2. Upload the Sample, Sequence, Subject and Manifest files to their corresponding consent-level buckets. For each unique upload, place files in a sub-bucket within each consent group bucket that reflects the **date of the upload** (e.g. "20210101", for January 1st, 2021). **If you do not yet have the bucket key for your site, please refer to the AnVIL Resources –> SFTP Server Access tab for instructions.** If you are transferring files from a normal server environment, you can use the gsutil tool. If you are transferring files from another cloud server, you may need to use an alternative data transfer protocol. Bucket access information for your site can be downloaded from the UW SFTP server.

3. Upload all BAMs, VCFs, index and BED files to their corresponding **consent-level dated sub-buckets.** The DCC has already created consent-level sub-buckets for each site, as shown in the diagram below.

4. Send an email to [redacted] with the following information (Note: the linked address will automatically include [redacted] in the recipient list):

   Subject Line: CSER [Site Name] Data Upload [YYYYMMDD]
   Email Body:
   * Total number of files of each type uploaded (vcf(.gz), vcf.tbi, bam, bam.bai, bed, tsv, README, etc.)
   * Number of files that were updated (already uploaded in a previous submission)
   * Any other upload details you would like to provide

DOWNLOAD ANVIL BUCKET PATHS FOR PARTICIPANTS AT YOUR SITE

Figure S9. Change log documentation on the CSER Data Hub website.

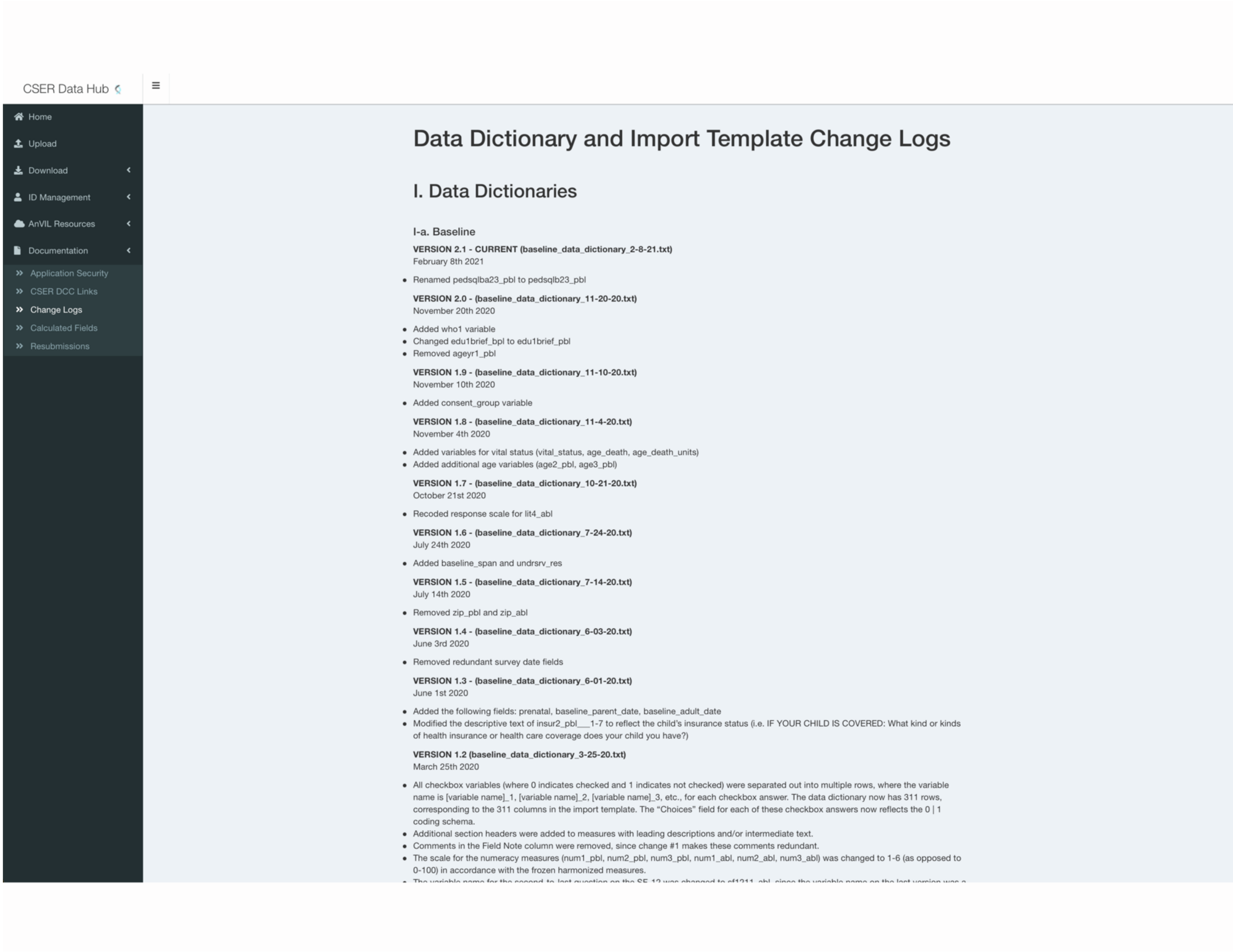

CSER Data Hub

- Home
- Upload
- Download
- ID Management
- AnVIL Resources
- Documentation
  - Application Security
  - CSER DCC Links
  - Change Logs
  - Calculated Fields
  - Resubmissions

Data Dictionary and Import Template Change Logs

I. Data Dictionaries

I-a. Baseline

VERSION 2.1 - CURRENT (baseline_data_dictionary_2-8-21.txt)
February 8th 2021

- Renamed pedsqlba23_pbl to pedsqlb23_pbl

VERSION 2.0 - (baseline_data_dictionary_11-20-20.txt)
November 20th 2020

- Added who1 variable
- Changed edu1brief_bpl to edu1brief_pbl
- Removed ageyr1_pbl

VERSION 1.9 - (baseline_data_dictionary_11-10-20.txt)
November 10th 2020

- Added consent_group variable

VERSION 1.8 - (baseline_data_dictionary_11-4-20.txt)
November 4th 2020

- Added variables for vital status (vital_status, age_death, age_death_units)
- Added additional age variables (age2_pbl, age3_pbl)

VERSION 1.7 - (baseline_data_dictionary_10-21-20.txt)
October 21st 2020

- Recoded response scale for lit4_abl

VERSION 1.6 - (baseline_data_dictionary_7-24-20.txt)
July 24th 2020

- Added baseline_span and undrsrv_res

VERSION 1.5 - (baseline_data_dictionary_7-14-20.txt)
July 14th 2020

- Removed zip_pbl and zip_abl

VERSION 1.4 - (baseline_data_dictionary_6-03-20.txt)
June 3rd 2020

- Removed redundant survey date fields

VERSION 1.3 - (baseline_data_dictionary_6-01-20.txt)
June 1st 2020

- Added the following fields: prenatal, baseline_parent_date, baseline_adult_date
- Modified the descriptive text of insur2_pbl___1-7 to reflect the child's insurance status (i.e. IF YOUR CHILD IS COVERED: What kind or kinds of health insurance or health care coverage does your child you have?)

VERSION 1.2 (baseline_data_dictionary_3-25-20.txt)
March 25th 2020

- All checkbox variables (where 0 indicates checked and 1 indicates not checked) were separated out into multiple rows, where the variable name is [variable name]_1, [variable name]_2, [variable name]_3, etc., for each checkbox answer. The data dictionary now has 311 rows, corresponding to the 311 columns in the import template. The "Choices" field for each of these checkbox answers now reflects the 0 | 1 coding schema.
- Additional section headers were added to measures with leading descriptions and/or intermediate text.
- Comments in the Field Note column were removed, since change #1 makes these comments redundant.
- The scale for the numeracy measures (num1_pbl, num2_pbl, num3_pbl, num1_abl, num2_abl, num3_abl) was changed to 1-6 (as opposed to 0-100) in accordance with the frozen harmonized measures.
- [illegible]

Figure S10. Reference sheet for Baseline Measures in the CSER cross-site Adaptation Dictionary.

BASELINE

	CHARM	Baylor	UCSF	HUDSONALPHA	Sinai/NYCKidSeq	NCGENES/UNC
Gender	A	A	A	D	SA	A
~~**DOB**~~						
Age year					SA	
Language	A	A	A	?	SA	A
Income	B	B			SA	
Education	B	B	B		SA	
Insurance	SA	SA	SA	SA	SA	
Country of origin	B	D?		D	A	D
Access	A			D	SA	
Literacy		A			SA	
Numeracy			D		SA	
Race/Ethnicity			D		SA	
R/E parent 1	NA		A			
R/E/parent 2	NA	D	A			D
~~**Zip**~~						
VAS			A	A		SA
SF-12		NA	NA	NA	NA	NA
PEDSQL	NA	D	D	D	SA	

**Legend**		**Notes**
Identical to harmonized item and response scale =		No changes to harmonized items or response scale
Identical to harmonized item and response scale. Brief version used =	B	Collapsed/Brief harmonized version used for this item
Slight Adaptation=	SA	Slight change in question, question format or response scale. Ex: changed formatting, slight changes to wording of question or response scale
Adaptation=	A	Significant change in question, question format or response scale. Ex: dropped or changed items, changed responses
Dropped=	D	Whole survey dropped
Removed from data base=		Items were removed due to changes in privacy policy
Scale not applicable to study population=		Ex: Adult scale in pediatric population

Table S1. Examples of modifications, additions, and transformations to the harmonized CSER survey measures and outcomes database.

See attached Excel spreadsheet.

Table S2. Harmonized sequence and sample metadata model.

See attached Excel spreadsheet.

Table S3. CSER harmonized consent groups. DUC = dbGaP Data Use Category; DUR = Data Use Restriction; GRU = General Research Use; HMB = Health/medical/biomedical research; IRB = Ethics Approval Required.

	**Survey Data**		**Sequence Data**	
**Consent Group**	**DUC**	**DUR**	**DUC**	**DUR**
1	GRU	None	GRU	None
2	GRU	IRB	GRU	IRB
3	GRU	IRB	N/A	CSER-ONLY Access
4	GRU	IRB	N/A	N/A
5	N/A	CSER-ONLY Access	GRU	IRB
6	N/A	CSER-ONLY Access	N/A	CSER-ONLY Access
7	N/A	CSER-ONLY Access	N/A	N/A
8	HMB	None	HMB	None
