# Supplementary material for: Lessons learned and recommendations for data coordination in collaborative research: The CSER consortium experience
Source: HGG Adv. 2022 May 20;3(3):100120. doi: 10.1016/j.xhgg.2022.100120 (PMC9190054; doi:10.1016/j.xhgg.2022.100120)
Supplement: Document S2. Article plus supplemental information [file mmc4.pdf]

HGG Advances

ARTICLE

Lessons learned and recommendations for data coordination in collaborative research: The CSER consortium experience

Kathleen D. Muenzen,[1,*] Laura M. Amendola,[2] Tia L. Kauffman,[3] Kathleen F. Mittendorf,[3] Jeannette T. Bensen,[4] Flavia Chen,[5] Richard Green,[1] Bradford C. Powell,[6] Mark Kvale,[5] Frank Angelo,[2] Laura Farnan,[7] Stephanie M. Fullerton,[8] Jill O. Robinson,[9] Tianran Li,[1] Priyanka Murali,[2] James M.J. Lawlor,[10] Jeffrey Ou,[2] Lucia A. Hindorff,[11] Gail P. Jarvik,[2] and David R. Crosslin[12,*]

Summary

Integrating data across heterogeneous research environments is a key challenge in multi-site, collaborative research projects. While it is important to allow for natural variation in data collection protocols across research sites, it is also important to achieve interoperability between datasets in order to reap the full benefits of collaborative work. However, there are few standards to guide the data coordination process from project conception to completion. In this paper, we describe the experiences of the Clinical Sequence Evidence-Generating Research (CSER) consortium Data Coordinating Center (DCC), which coordinated harmonized survey and genomic sequencing data from seven clinical research sites from 2020 to 2022. Using input from multiple consortium working groups and from CSER leadership, we first identify 14 lessons learned from CSER in the categories of communication, harmonization, informatics, compliance, and analytics. We then distill these lessons learned into 11 recommendations for future research consortia in the areas of planning, communication, informatics, and analytics. We recommend that planning and budgeting for data coordination activities occur as early as possible during consortium conceptualization and development to minimize downstream complications. We also find that clear, reciprocal, and continuous communication between consortium stakeholders and the DCC is equally important to maintaining a secure and centralized informatics ecosystem for pooling data. Finally, we discuss the importance of actively interrogating current approaches to data governance, particularly for research studies that straddle the research-clinical divide.

Introduction

In the burgeoning field of medical genetics, aggregating and sharing data across research settings and clinical environments is key to expanding the evidence base for clinical genetic testing and for increasing the generalizability of research findings to diverse clinical settings.[1] Collaborative, multi-site research consortia help address a critical need for high-quality, shared clinical research data by providing access to a broad range of patient populations, care environments, and shared resources for data analysis.[2] However, multi-site collaborations pose significant challenges for sharing and managing research data, such as patient and research participant privacy considerations, data harmonization challenges, and complex communication requirements.[3] Amidst ongoing debates surrounding how best to share research data, recent National Institutes of Health (NIH) initiatives, like the Genomic Data Sharing (GDS) Policy, have required that data sharing become a standard practice in genomics research.[4] While the NIH GDS Policy requires all NIH-funded research projects that generate large-scale genomic data to share their data and associated metadata for use in future research, it does not specify how data sharing should be implemented. Despite the ubiquity of data sharing requirements in the field of genomics, standard best practices for managing multi-site datasets and articulating and mitigating potential risks to participants have yet to be widely developed, adopted, or implemented.[5,6]

To address the need for effective data coordination across multiple institutions, Data Coordinating Centers (DCCs) are often established within research consortia to facilitate data sharing. Large projects, like the Electronic Medical Records and Genomics (eMERGE) Network, the Alzheimer's Disease Research Centers (ADRCs), the Digitalis Investigation Group (DIG) trial, the model organism Encyclopedia of DNA Elements (modENCODE) project, and the Li-Fraumeni Exploration (LiFE) Consortium, have implemented DCCs to facilitate data sharing and integration across heterogeneous research environments.[7–11] Projects that share

[1]Department of Biomedical Informatics and Medical Education, Division of Biomedical and Health Informatics, University of Washington Medical Center, Seattle, WA, USA; [2]Department of Medicine (Medical Genetics), University of Washington Medical Center, Seattle, WA, USA; [3]Center for Health Research, Kaiser Permanente Northwest, Portland, OR, USA; [4]Department of Epidemiology, Gillings School of Global Public Health, University of North Carolina at Chapel Hill, Chapel Hill, NC, USA; [5]Institute for Human Genetics, University of California at San Francisco, San Francisco, CA, USA; [6]Department of Genetics, University of North Carolina at Chapel Hill, Chapel Hill, NC, USA; [7]Lineberger Comprehensive Cancer Center, UNC School of Medicine, University of North Carolina at Chapel Hill, Chapel Hill, NC, USA; [8]Department of Bioethics & Humanities, University of Washington School of Medicine, Seattle, WA, USA; [9]Center for Medical Ethics and Health Policy, Baylor College of Medicine, Houston, TX, USA; [10]HudsonAlpha Institute for Biotechnology, Huntsville, AL, USA; [11]Division of Genomic Medicine, NHGRI, NIH, Bethesda, MD, USA; [12]Division of Biomedical Informatics and Genomics, John W. Deming Department of Medicine, Tulane University School of Medicine, New Orleans, LA, USA
*Correspondence: kmuenzen@uw.edu (K.D.M.), crosslin@tulane.edu (D.R.C.)
https://doi.org/10.1016/j.xhgg.2022.100120.
© 2022 The Author(s). This is an open access article under the CC BY license (http://creativecommons.org/licenses/by/4.0/).

large-scale genomic data must also consider the rapidly evolving landscape of data security and data standards in genomics, in addition to the logistical challenges of integrating heterogeneous datasets.[6,12]

The Clinical Sequence Evidence-Generating Research (CSER) Consortium is a multi-site program funded by the National Human Genome Research institute (NHGRI), National Cancer Institute (NCI), and National Institute on Minority Health and Health Disparities (NIMHD) and has been navigating the complexities of data sharing across a network of projects. In its second phase of funding, CSER is investigating the effectiveness of integrating genomic sequencing into clinical care, particularly in diverse and medically underserved populations.[13] Recognizing the administrative challenges posed by data coordination as consortium members worked to identify and implement harmonized survey measures across participating research sites,[14] the CSER DCC was funded 2 years into this second phase of the CSER program and worked to aggregate harmonized survey and sequence data across six extramural projects and one NHGRI intramural project. The primary purpose of the CSER DCC was to design, develop, test, and deploy the infrastructure to aggregate harmonized survey and genomic sequencing data in a secure and centralized way.

As of March 2022, over 55% of the planned consortium manuscripts intended to use harmonized measures and/or case-level sequencing metrics data.[15] These planned manuscripts covered a wide array of topics, such as family sharing of genomic sequencing results, patient satisfaction with result disclosure, information-seeking behaviors among research participants and family members, and overall perceptions of the clinical utility of genome sequencing. They collectively aimed to address the multidimensional challenges posed by genome-wide clinical sequencing while assessing the clinical and personal utility of genomic medicine. Eighteen percent of planned manuscripts intended to use centrally shared genomic sequence data to assess methods for determining genetic ancestry groups, search for novel disease-causing variants, and contribute to data analysis standards for clinical sequencing labs.

In this paper, we first describe the CSER consortium and its data coordination needs and then describe the consortium's experience with implementing a DCC to manage heterogeneous survey, phenotypic, and genomic data across sites. Using our own successes, opportunities for growth, and lessons learned as a guide, we offer a set of recommendations for other research consortia to consider when designing data coordination plans for multi-site collaborative projects, particularly in the field of clinical genomics.

Materials and methods

Consortium structure and communication

CSER consisted of a Steering Committee and eight main working groups with members from the following contact institutions and CSER projects: (1) Baylor College of Medicine (KidsCanSeq), (2) Kaiser Permanente Northwest (CHARM), (3) University of North Carolina at Chapel Hill (NCGENES 2), (4) Icahn School of Medicine at Mount Sinai (NYCKidSeq), (5) University of California, San Francisco (P^3EGS), (6) HudsonAlpha Institute for Biotechnology (SouthSeq), and (7) The National Human Genome Research Institute (ClinSeq). Consortium activities were facilitated by a coordinating center based at the University of Washington and were guided by an external committee, the CSER Advisory Panel, consisting of six experts in genomic medicine and a community advocate. While all CSER sites shared a common goal of investigating the applications and outcomes of genomic sequencing in clinical care, the patient populations, specific research aims, and study protocols differed widely between sites (Figure S1). Detailed descriptions of CSER working groups, study populations, and sequencing methodologies are described in Amendola et al.[13] and Goddard et al.[14]

Consortium communication was facilitated through monthly working group video calls, biweekly coordinating center calls, monthly Steering Committee calls, and tri-annual consortium-wide meetings. At the start of the COVID-19 pandemic in early 2020, communications became entirely virtual. The DCC interacted extensively with the Data Wranglers working group (established by the DCC in Fall 2019) and the Project Managers working group (established in Spring 2019). Interactions largely consisted of monthly video calls and ad hoc calls with individual site analysts and project managers.

The DCC collaborated with several external organizations that helped maintain the technical infrastructure that the consortium used to securely manage its aggregated survey and sequence data. The Institute of Translational Health Sciences (ITHS) at the University of Washington managed the Research Electronic Data Capture (REDCap) database[16,17] that the DCC used for centralized CSER data storage and maintained a secure web server that hosted the consortium's R Shiny[18] data management tool. The DCC also collaborated extensively with the NHGRI Genomic Data Science Analysis, Visualization, and Informatics Lab Space (AnVIL) consortium, which was responsible for hosting shared CSER genomic, clinical, survey, and phenotypic data in the AnVIL cloud computing ecosystem.[19]

Timeline of CSER data harmonization, collection, and analysis activities

The second phase of CSER began in August 2017. Harmonized measures were developed throughout 2018, and sites adopted the harmonized measures in late 2018. As described in Goddard et al.,[14] sites designed most of their data collection instruments independently and began recruitment and/or survey administration up to 18 months after the consortium start date. By the time the consortium had finalized the harmonized measures in late 2018, several sites had already begun administering surveys and were tasked with administering some harmonized items that they had not previously implemented. The DCC developed the initial harmonized database and custom data collection platform throughout the fall and winter of 2019–2020. The DCC began coordinating the centralized intake of common survey measure responses in early 2020 and continued to collect these data until the end of the recruitment and follow-up periods at each site. Initial requests for—and preliminary analysis of—harmonized survey data began in fall 2020, and the first submissions of genome and exome data to the AnVIL cloud platform began in

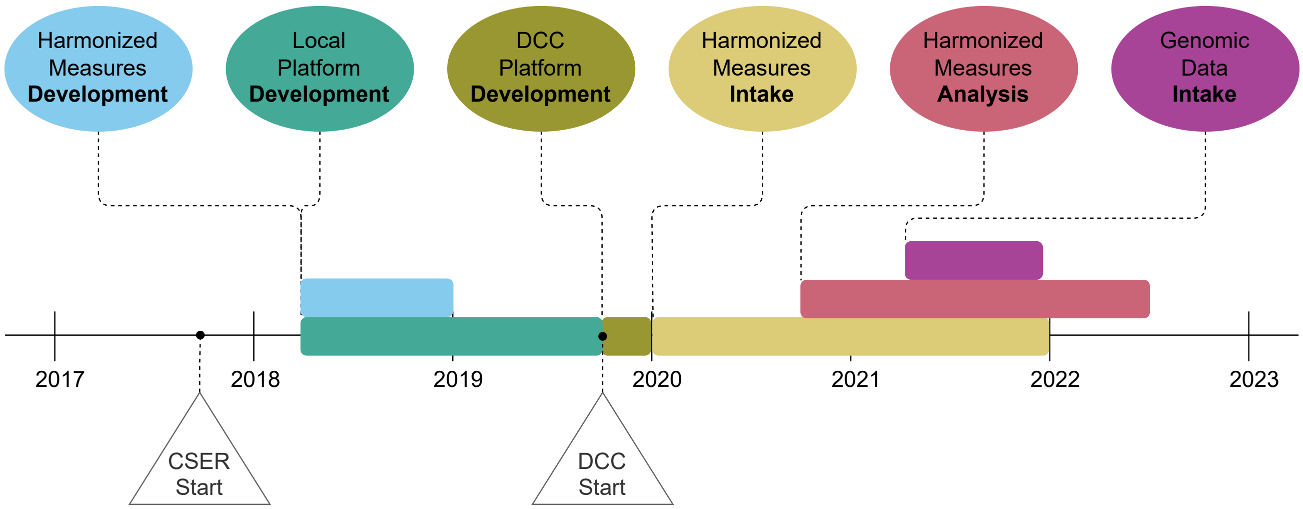

Figure 1. CSER phase 2 data coordination and analysis timeline

spring 2021, shortly after the AnVIL platform was designated as an official NIH data repository.[20] A timeline of major consortium-wide activities related to data harmonization, collection, and analysis is shown in Figure 1.

Informatics architecture

The DCC utilized a suite of informatics tools and platforms to securely store and share consortium data. The following tools and platforms were used to coordinate CSER data.

Local site servers and data capture tools

Data was collected and stored locally by each CSER site before it reached the DCC. Sites collected survey data using platforms including REDCap, SurveyMonkey, and custom-developed web applications. Some measures (like participant ages) were pulled directly from the Electronic Health Record (EHR) by sites if they were not collected through harmonized surveys. Methods for survey data storage also varied by site, with some sites using REDCap databases or similar platforms designed for clinical research and others using relational or non-relational database-management systems for optimized storage and querying of large datasets. The vast majority of survey data quality assurance (QA) and quality control (QC) was performed at CSER sites prior to DCC submission. These QA/QC measures included, but were not limited to, checks for missing data, range value checks, and outlier analyses. Genomic data were stored on servers with high disk capacity at each site or using secure cloud storage services like Amazon S3 or Microsoft Azure.

REDCap database

A secure instance of REDCap was hosted and maintained by the University of Washington ITHS and populated by CSER sites using data-submission tools maintained by the DCC. All harmonized survey measures, case-level sequencing results, and participant-level sequencing metrics (e.g., aggregated case-level results) were centrally stored in REDCap and were linked at the participant level using a unique identifier called a "CSER ID."

CSER Data Hub

The DCC used a custom R Shiny web interface called the "Data Hub" to securely exchange harmonized survey data, case- and participant-level sequencing metrics, and documentation within the consortium. See "informatics" and "data de-identification and security" for more details on the architecture and security features of the Data Hub.

AnVIL storage and compute platform

The NIH-funded AnVIL consortium develops and maintains the AnVIL cloud ecosystem, which was built using Google Cloud storage and compute resources. The AnVIL is a component of the emerging federated data ecosystem paradigm in genomics,[21] which is meant to improve genomic data sharing and interoperability without compromising data security or privacy. The AnVIL is authorized to share both open-access (unrestricted) and controlled-access (restricted) data derived from human samples.[20] Permission to access and use controlled-access data is granted on a case-by-case basis by a relevant NIH Data Access committee and is moderated through the database of Genotypes and Phenotypes (dbGaP) Authorized Access System.[22] CSER sites were required to submit their genomic binary alignment map (BAM) and variant call format (VCF) files, sequence and sample metadata (e.g., reference genome build and sample source), and phenotypic data (e.g., disease codes, sex, and race or ethnicity) to the AnVIL platform. Data stored in the AnVIL could then be analyzed in Terra,[23] a cloud platform developed by the Broad Institute of MIT and Harvard to facilitate biomedical research data sharing and analysis.

Collection and aggregation of harmonized survey measures

To collect common survey measures administered at each site, the DCC developed a REDCap database using the harmonized survey measures developed by the consortium in 2018[15] and worked with the Data Wranglers working group to map site-specific data models to a harmonized data model using a three-phase approach.

Phase 1: Model

To facilitate mapping between site datasets and the DCC harmonized database, the DCC developed tab-delimited import templates and accompanying data dictionaries for six harmonized survey types (Figure S2). All patient surveys were divided into two distinct variable sets to distinguish between surveys administered to a parent or guardian proxy of a pediatric participant and those administered to an adult participant. The DCC also developed standardized import templates and data dictionaries for participant-level and case-level genetic sequencing metrics (Figure S3). All templates and data dictionaries were distributed as downloadable zip files on the Data Hub.

Table 1. Data coordination lessons learned in the CSER consortium

Category	Lessons learned
Communication	1a. Identify primary points of contact for addressing different data coordination requirements (e.g., technical infrastructure, data mapping, and consortium policy) using existing communication patterns among working groups and sites<br>1b. Define the unique roles of different working groups in the data coordination process and use those roles to guide inter-group communication<br>1c. Send periodic update emails with consolidated information (progress, resources, and action items) to key data coordination stakeholders
Harmonization	2a. Provide data managers with standardized data collection instruments (templates) and specifications for mapping variables to those instruments (data dictionaries)<br>2b. Deploy rigorous version-control methods for data coordination resources that change over time and ensure that data managers are informed of changes<br>2c. Implement standardized protocols and timelines for making changes to data collection instruments<br>2d. Engage a multidisciplinary group of consortium members to develop and approve standardized data models
Informatics	3a. Consolidate informatics tools and resources within a secure, centralized platform<br>3b. Utilize available information technology (IT) expertise and resources at participating institutions<br>3c. Prioritize security of informatics tools and disseminate security information to consortium members
Compliance	4a. Engage a multidisciplinary group of consortium members to develop a harmonized set of data sharing consent categories<br>4b. Use multiple data-sharing specifications (e.g., institutional certifications, informed consents, and data use letters) to map site-level consent groups to consortium-level consent categories
Analytics	5a. Document data-quality issues and unique aspects of the harmonized dataset and plan to distribute documentation to both current and future data users<br>5b. Facilitate access to onboarding resources for users of shared data analysis platforms like the AnVIL

Phase 2: Map

Site analysts developed semi-automated variable mapping pipelines using the data-handling software(s) of their choice (e.g., Excel, R, Python, Stata, and SAS) and used these pipelines to generate harmonized datasets from the harmonized data model developed in phase 1.

Phase 3: Upload

Staff at each site shared their harmonized datasets through a custom data-upload interface on the Data Hub, which ensured that the datasets met the specifications of the models developed during phase 1, and automatically transferred data to the DCC REDCap database using the redcapAPI R package.[24] Initial submissions for each of the harmonized survey types and sequencing metrics occurred in 2- to 3-month intervals throughout 2020 and 2021.

All sites repeated phases 2 and 3 on a quarterly basis until the end of follow-up to update existing participant records, and to create records for newly recruited participants.

Genomic sequence data collection in the AnVIL

The CSER DCC facilitated the transfer of genome and exome data and metadata from site platforms to the AnVIL platform. The DCC developed harmonized metadata models in collaboration with members of the AnVIL team and other CSER members, using standards previously developed by dbGaP and The Cancer Genome Atlas (TCGA) Program as references. To facilitate the transfer of sequence data and metadata to the AnVIL platform, the DCC developed sample scripts for securely transferring data to Google Cloud buckets and made these scripts available for download on an SFTP server hosted by the University of Washington Genome Sciences department. The DCC also provided step-by-step instructions for preparing data, submitting required data ingest forms, and using sample scripts for batch sequence data transfers.

Results

Lessons learned

Throughout 2020 and 2021, the DCC worked to meet the evolving data coordination needs of the CSER consortium as it actively collected sequence and survey data from study participants. The following section describes the approaches that the CSER consortium used to navigate the complexities of multi-site data sharing and offers a set of lessons learned from its data coordination experiences (Table 1). Lessons learned are referenced in the text using numbered identifiers (e.g., lesson learned 1a, lesson learned 1b) to exemplify connections between experiences and lessons learned.

Communication

As the DCC integrated with the consortium throughout 2020, additional communication channels beyond monthly Data Wranglers working group calls were formed to fully support the consortium's data coordination requirements. While the Data Wranglers primarily served the role of handling site-level survey and sequence data and developing computational pipelines to convert data into a harmonized format, the Project Managers provided the necessary project-level guidance to ensure that data were being shared securely and responsibly, such as tracking regulatory documents, overseeing data collection, and developing data QA/QC measures. Together, the two working groups contributed to the development of feasible and efficient DCC harmonized data-upload requests and data dictionaries, assisted in coordinating responses to new data

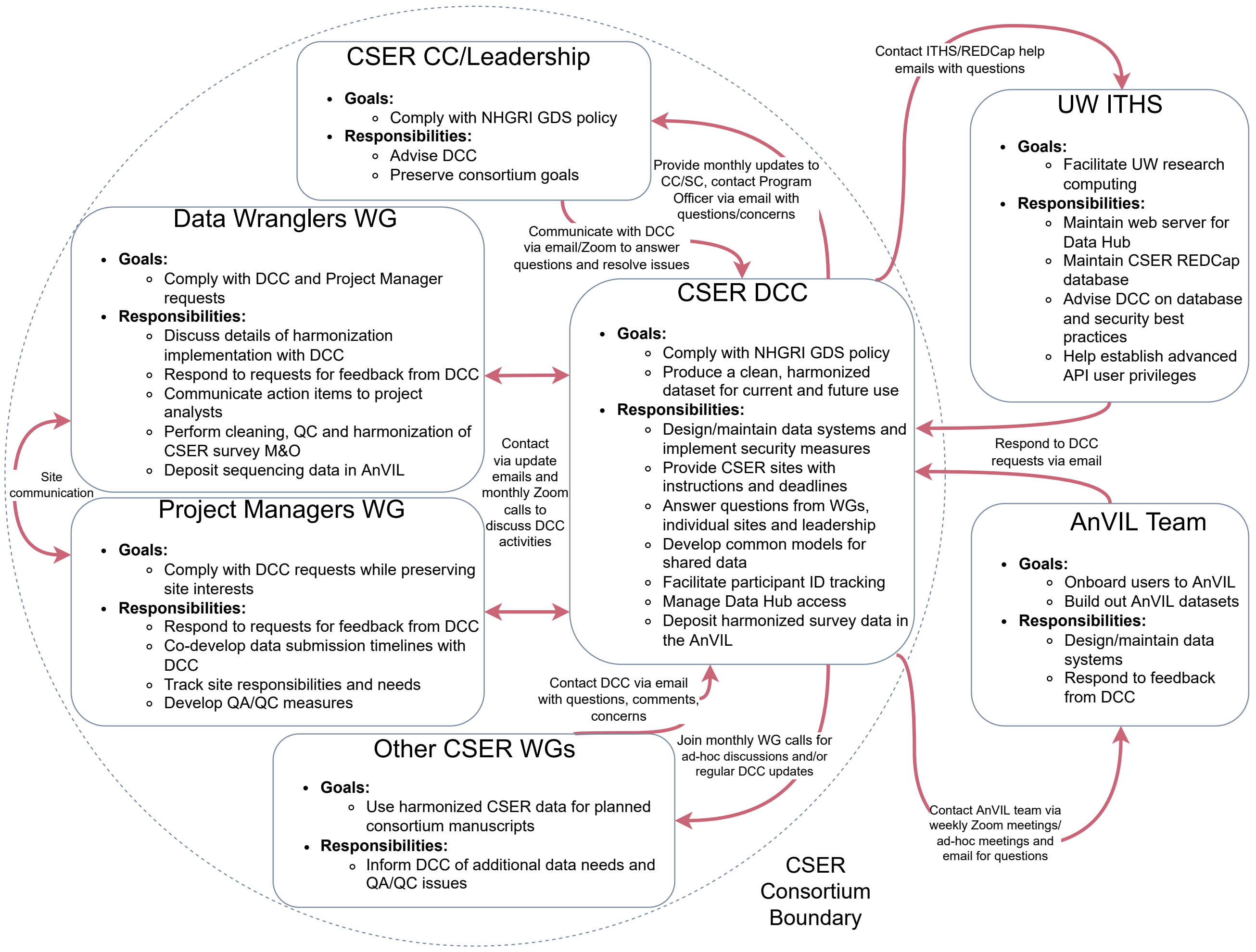

Figure 2. Methods of communication between groups involved in CSER data coordination

requests (including site-specific data), assisted in troubleshooting challenging data elements (e.g., consent categories), responded to requests for project-specific information, and kept track of data submission timelines (lesson learned 1a). The DCC, Data Wranglers, and Project Managers communicated through an iterative, multi-directional feedback loop throughout the project period to ensure that all groups were equipped to fulfill their respective data coordination responsibilities (lesson learned 1b).

Multiple working groups requested that the DCC share important data coordination updates with the rest of the consortium. To increase transparency of ongoing work and maintain an organized list of action items, the DCC sent update emails to the Data Wranglers working group, Project Managers working group, Sequence Analysis and Diagnostic Yield working group, and principal investigators (PIs), first on a biweekly and eventually on a monthly basis to communicate important DCC activities, inform consortium members of key resources, and track new data coordination requirements. To communicate DCC activities and goals with the broader consortium, the DCC also gave regular progress updates during biweekly and monthly Coordinating Center and Steering Committee calls, respectively. These updates helped other working groups and consortium stakeholders anticipate availability of shared data and allowed consortium members outside of the Project Managers, Data Wranglers, and Sequence Analysis and Diagnostic Yield working groups to regularly provide feedback and ask questions about current and planned DCC initiatives (lesson learned 1c).

Interactions between the DCC and groups external to the consortium were largely facilitated by weekly or biweekly standing meetings, including those with AnVIL project managers and the University of Washington ITHS staff. These meetings helped the DCC receive timely assistance and feedback from technical support teams and to communicate questions and concerns raised by CSER members. Figure 2 shows the different groups involved in CSER data coordination, their responsibilities, and the types of communication that took place between different stakeholders.

Harmonization

Survey data harmonization

Throughout 2020 and 2021, the DCC developed a variety of strategies to facilitate the harmonization and intake

of common survey measures. As described in Goddard et al.,[14] the CSER Measures and Outcomes Working Group previously led the consortium through identifying 31 survey domains across CSER projects that captured measures related to the common research aim of evaluating the personal and clinical utility of genome and exome sequencing while accommodating natural heterogeneity in study designs and patient populations. Common survey measures were presented to research participants in a wide variety of study environments, altered to meet the needs of individual sites, and collected and stored using different data-modeling strategies. As a result, measures were harmonized across many factors, including question wording, response scales, and variable naming. While measure harmonization was important for achieving cross-site interoperability of research findings, it was also a time-consuming effort that required careful planning and use of limited resources.

In CSER's experience, achieving and sharing semantically interoperable data was far more complex than simply sharing data. As described in "consortium structure and communication," the seven CSER projects served different patient populations, investigated unique research questions, and used different clinical sequencing interventions (Figure S1). Furthermore, sites developed their own data collection tools before a clear set of centralized data-sharing expectations was established. To reconcile differences between site-specific implementations of common survey measures, the DCC developed standardized data import templates and data dictionaries to guide harmonized survey mapping, as described in "collection and aggregation of harmonized survey measures" (lesson learned 2a). The complexity of this process is illustrated in Figure 3, which depicts the mapping process for a single variable in the communication satisfaction measure from the first Patient Post-Return of Results (RoR) survey. By the end of the survey-mapping phase for all six harmonized surveys and two sequencing metric reports, sites had implemented mapping logic for over 1,100 variables.

The primary goal of the survey mapping phase (phase 2) was for each site to develop a semi-automated pipeline that could be used to quickly update harmonized datasets with new or modified data on a quarterly basis. However, the pipeline development process was complex and time intensive for each site and involved frequent updates to mapping logic. Updates included relatively simple changes, like variable name modifications and harmonized response scale adjustments, but also included more complex updates, like the addition of new variables that were deemed necessary for accurate, reliable, and secure downstream analysis of harmonized data (Table S1). For example, the elapsed time since RoR variable was first proposed during a Data Wranglers working group meeting in July 2020, when it was discovered that not all participant or provider follow-up surveys could be administered or collected within the harmonized time frames specified (Figure S2) and that having more granular elapsed time data could improve the accuracy of downstream analyses. A placeholder variable was developed and then iteratively refined before seeking Steering Committee and Institutional Review Board (IRB) approval. The finalized variable required sites to indicate the number of weeks post-RoR that a given survey or measure was administered to each participant. Sites were then tasked with implementing new mapping logic for as few as three and as many as 25 new harmonized variables, depending on whether follow-up measures were administered according to the harmonized survey groups (Figure S4). While not all change requests were this lengthy or involved, they cumulatively resulted in high demands on Data Wranglers and Project Managers throughout the harmonized measure mapping process.

To minimize burden placed on Data Wranglers and Project Managers due to change requests and to maximize transparency, the DCC maintained a "change log" page in the Data Hub, which listed the changes made between import template and data dictionary versions. During the last quarter of 2020, the DCC began distributing quarterly checklists that documented all new, removed, and modified variables for each quarterly data resubmission and made these documents available for download on the Data Hub (lesson learned 2b). Beginning in January 2021, the DCC also implemented a new "change request schedule," which specified time intervals when consortium members could make change requests and blocked off 2-month intervals before each quarterly resubmission, during which site data analysts could modify mapping pipelines without having to address incoming change requests. These strategies helped manage the computational and organizational burden of maintaining harmonized mapping pipelines but nonetheless did not eliminate all tensions between site-level burden and consortium-level data-sharing expectations (lesson learned 2c).

Sequence metadata harmonization

The AnVIL replaced dbGaP as the primary repository for NHGRI-funded genomic, phenotypic, and survey datasets in mid-2019, during the CSER phase II funding period.[20] While dbGaP provided data submitters with standardized templates and instructions for submitting sequence data and metadata to the platform, the AnVIL consortium was still developing standards when CSER commenced submissions. As a result, the CSER DCC was tasked with developing standardized metadata models that captured the necessary details without placing unreasonable burden on CSER sites. In mid-2020, the DCC convened a subgroup of CSER investigators (called the "Sequence Metadata Subgroup") with expertise in sequence data analysis to develop a harmonized set of sequence and sample metadata fields (lesson learned 2d). Prior to the first subgroup meeting, the DCC compiled a list of candidate variables using a combination of the dbGaP and TCGA standards. The DCC presented these variables to the Sequence Metadata Subgroup to assess the feasibility and descriptiveness of the proposed fields. Once the model was approved by the

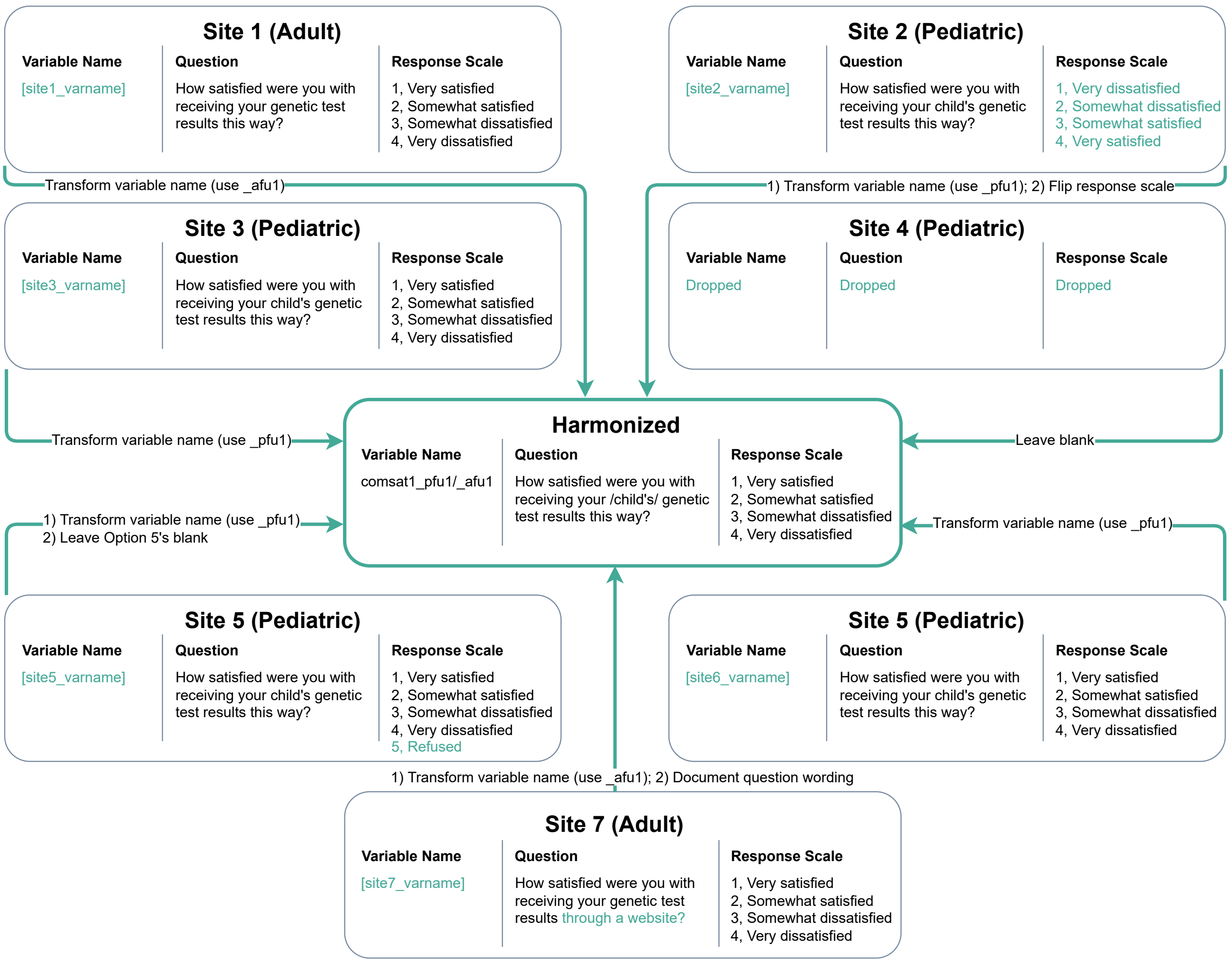

Figure 3. Sample harmonization process for one variable in the communication satisfaction measure, across all seven CSER projects
To map participant responses to the Participant Post-Return of Results (RoR) Follow-Up no. 1 harmonized import template, each site created a local mapping between the site-level variable name and the harmonized variable name (comsat1_pfu1 for pediatric surveys and comsat1_afu1 for adult surveys) and documented any changes in question wording. Some sites were also required to map alternate response encodings to the harmonized response scale. For example, site 2 administered the question with a reversed response scale (where 1 is "very satisfied" on the harmonized scale and 4 is "very satisfied" on the site scale) and modified harmonized responses accordingly (1 = 4, 2 = 3, 3 = 2, and 4 = 1). Similarly, site 5 administered the question with an additional response option and was instructed to map these responses to blank values (5 = '').

Sequence Metadata Subgroup, the Data Wranglers working group, and the AnVIL team, the DCC developed the relevant import templates and data dictionaries and made these documents available for download on the Data Hub (Table S2).

Informatics

The CSER DCC used the Data Hub platform to host data coordination resources in a centralized, secure, and easily accessible location. The Data Hub made it possible to link multiple data-management platforms with one another (Figure 4) and to quickly distribute version-controlled resources to Data Wranglers and Project Managers (lesson learned 3a). To develop and maintain the Data Hub, the DCC harnessed available information technology expertise and resources at the University of Washington ITHS (lesson learned 3b). However, they also relied heavily on informatics expertise within the DCC to develop the application itself and to provide troubleshooting support to CSER sites. Sample screenshots of the Data Hub user interface are shown in Figures S5–S9.

Data de-identification and security

Before submitting harmonized data to the Data Hub or sequence data to the AnVIL, all CSER sites were required to remove personally identifiable information (PII) from their datasets in accordance with the Health Insurance Portability and Accountability Act (HIPAA) Privacy Rule.[25] To retain syntactic integrity of free text, sites were asked to redact all instances of PII and replace them with the category of identifier within brackets (e.g., "[date]" and "[name]"). Measures were also taken to protect local

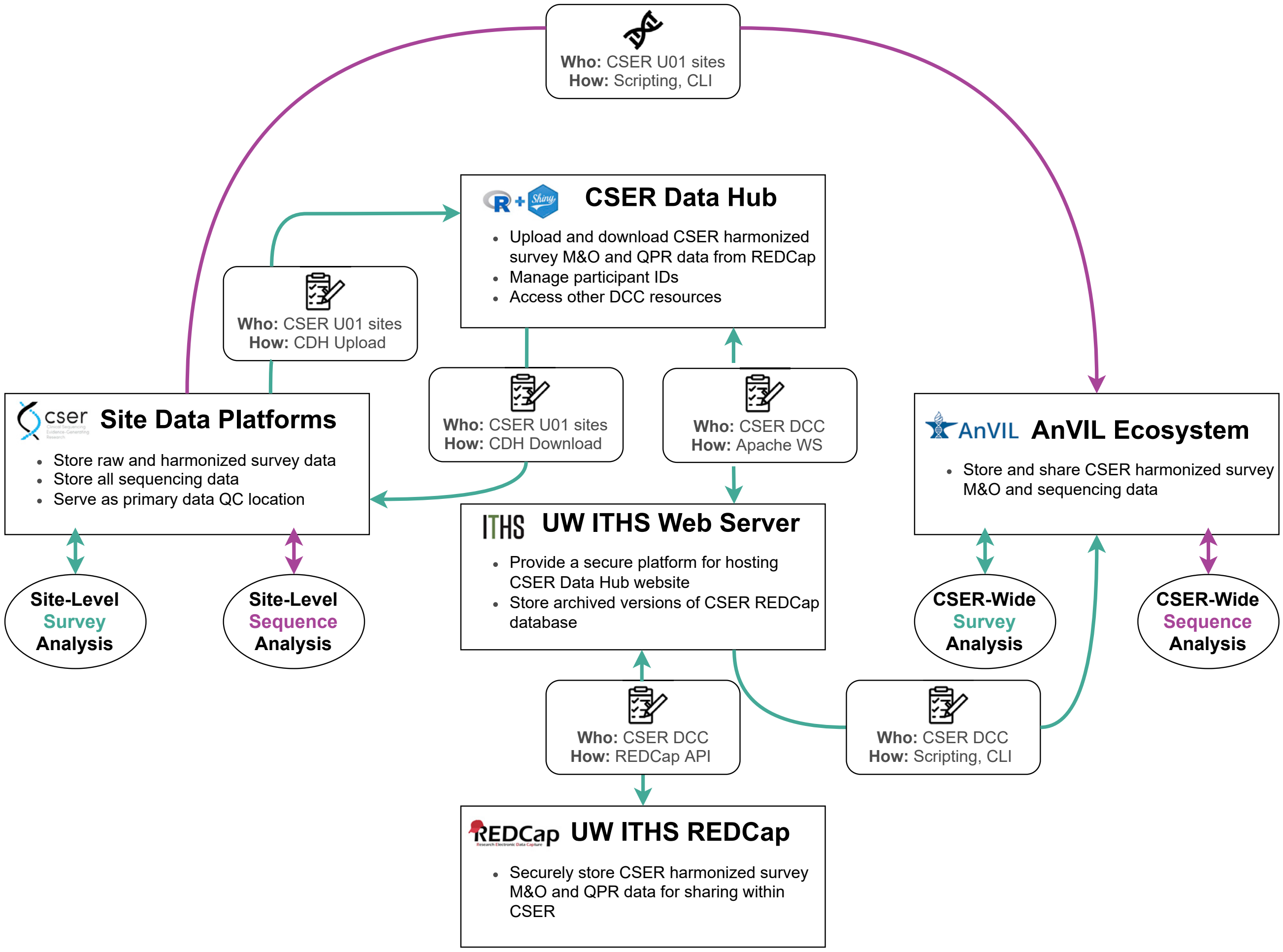

Figure 4. Movement of harmonized survey data (green) and sequence data (purple) between CSER data platforms
CDH, CSER Data Hub; CLI, command line interface; DCC, Data Coordinating Center; M&O, measures and outcomes; QPR, quarterly progress report; WS, web services.

study identifiers for each participant. For each new record in the harmonized database, a unique CSER ID was randomly generated and linked with the participant's local study ID. Mappings between CSER IDs and local IDs were then stored within the DCC REDCap database, accessible only to members of the site from which each CSER ID originated.

Although the DCC took steps to prevent identifiable information from being uploaded to its platforms, multiple layers of security were built into the DCC informatics architecture to protect data in the unlikely event that sensitive, identifiable information was to be uploaded to a DCC platform (lesson learned 3c). First, the Data Hub was deployed on a secure web server hosted by the University of Washington ITHS. All requested connections from client web browsers were established using the Apache HTTP Server software, and ITHS required that all hosted web applications establish encrypted connections between the server and the client browser. Second, all Data Hub users were required to log in to the Data Hub using University of Washington credentials, which were sponsored by the DCC team. Third, the Data Hub was designed in alignment with standards put forth by the HIPAA Security Rule, including the use of activity logs, password-protected access, automatic password timeout, and HIPAA-compliant data storage in REDCap. And fourth, the DCC developed standard protocols for removing records of participants that had withdrawn consent for sharing data and continuously updated and distributed a list of CSER IDs that should be removed from previously downloaded datasets.

Consent group harmonization

CSER did not have a central study IRB and thus relied on IRBs at each CSER site (and in some cases additional IRBs at subsites) and the University of Washington—the Coordinating Center home institution—to make decisions about appropriate data sharing. All site and Coordinating Center PIs signed a Data Use Agreement in early 2019 detailing the data-sharing terms between participating institutions in CSER, and the DCC used this document to broadly define the terms of data sharing across CSER sites and beyond the consortium.

While the use of local IRBs facilitated the implementation of varied clinical study designs across diverse patient populations at each site, the lack of a central CSER IRB also resulted

in substantial heterogeneity in how data-sharing consent groups were defined across CSER sites. Because the dbGaP Authorized Access System typically inherits consent group specifications from study institutional certifications,[26] the DCC first surveyed all institutional certifications to determine whether they sufficiently represented site-level consent groups. Following conversations with the CSER Project Managers, the DCC determined that while the institutional certifications provided high-level guidelines for how study data could be shared with non-CSER investigators, they did not fully represent subtleties of the permissions given by participants for sequence and/or survey data sharing during informed consent. For example, several CSER sites allowed participants to opt out of broad data sharing (e.g., general research use or health/medical/biomedical research) and to restrict sharing to specified investigators, while other sites required study participants to consent to broad data sharing if they were to enroll in the study. As a result, harmonized consent categories had yet to be developed when CSER sites were otherwise ready to share data.

To develop consortium-wide data sharing consent categories, the DCC convened a multidisciplinary "Data Access Subgroup" of data analysts, project managers, and data ethicists to discuss key considerations and requirements for consent harmonization (lesson learned 4a). The subgroup met twice over a period of 2 months in mid-2020 to develop a plan for mapping site-level consent categories to harmonized consent groups. Using a combination of standard NIH consent groups (e.g., general research use and health/medical/biomedical research) and data use limitations (e.g., local IRB approval required and publication required)[27] indicated in the site institutional certifications and more restrictive data-use limitations gleaned from site-specific informed consents (e.g. CSER-only access), the Data Access Subgroup developed eight harmonized consent groups for survey and sequence data types (Table S3; lesson learned 4b).[28] The Project Managers and Data Wranglers mapped participant-level consent groups to harmonized consent groups and submitted these consent assignments to the Data Hub in early 2021. These groups were used to determine how sequence and survey data could be stored and shared with non-CSER investigators in the AnVIL platform.

Cloud data sharing

The movement of data storage and computation to cloud platforms like Google Cloud, Amazon Web Services (AWS), or Microsoft Azure is widely regarded as a necessary next step in the field of genomics, given the large volume of genomic data generated daily, the increasing sophistication and scalability of cloud resources, and the need for extensive collaboration in genomic research.[29] While the goal of this transition is to maximize the utility and impact of human-derived samples and phenotypic data, cloud technology is still relatively novel to most academic institutions—which have historically used privately managed, secure servers to store and process genomic data—and to many research participants contemplating broad data sharing. While the NIH has previously released guidance on best practices for cloud data sharing,[30] the technical aspects of data security and administrative aspects of data privacy in the cloud are evolving. As a result, many institutions approach new cloud data-sharing requirements with caution.[31] The CSER consortium responded to cloud data-sharing requirements by reviewing informed consent documents at each site and ensuring that research participants gave their consent to share data in NIH controlled-access repositories other than dbGaP. The DCC also collaborated with the AnVIL team to compile security documentation into a single resource that sites could use to personally assess the security of datasets submitted to the platform, particularly those restricted to use within the consortium. Consistent communication between the AnVIL team, NIH staff, the DCC, and CSER working groups was essential for building consortium-wide trust in this new technology and for ensuring the ongoing privacy and security of de-identified genomic, phenotypic, and survey data in the new era of cloud storage and computing.

Analytics and documentation

Harmonized survey data reliability

Given the heterogeneity in how common survey measures were modeled and administered at each CSER site, the DCC developed strategies to document differences in site-level measure implementations. The DCC initially used separate Google Sheet data dictionaries for each site to document unique implementations of common measures. These site-level data dictionaries were then compiled into a single "adaptation dictionary," which documented the adaptations made to each harmonized variable across all CSER sites and was designed to highlight the degree to which each measure might be subject to data integration or reliability issues during analysis. To facilitate quick assessments of data reliability, the DCC implemented a cover sheet within the adaptation dictionary that indicated to what extent each measure was adapted (Figure S10). Step-by-step instructions were also included on the first tab of the dictionary to help investigators consider how adaptations might affect their analyses. To increase adoption within CSER, the DCC provided a link to the adaptation dictionary on the Data Hub and advised CSER members to reference the dictionary before attempting any cross-site analyses. The adaptation dictionary was intended for use by investigators both within and beyond CSER and was designed to be shared on platforms like the AnVIL to enhance the usability of CSER data for future research.

In addition to documenting adaptations to harmonized measures, the DCC developed a centralized help document for current and future users of CSER data. The document contained descriptions of all CSER projects, explanations for how key variables were harmonized, rationale for and descriptions of items that were added to the harmonized measures (e.g., vital status and survey completion dates),

and frequently asked questions (FAQs) related to database structure and use (lesson learned 5a).

The DCC also implemented several automated, on-demand variable calculation features in the Data Hub to generate measures that could be programmatically derived from the harmonized measures. The CSER "Underserved Framework," developed by members of the CSER Ethical, Legal, and Social Implications and Diversity working group, employed different combinations of demographic factors (including language, income, insurance status, residence, race, and ethnicity) to form nine distinct risk groups, indicating either direct barriers to medical care access or social factors that might indirectly impede access. Using the Data Hub download tool, consortium members could elect to download automatically calculated Underserved Framework variables along with documentation about how each variable was calculated.

Using the AnVIL platform for analysis

The AnVIL platform seeks to enable users with scalable compute power, large-scale data access, and shared resources for analysis.[19] The AnVIL analysis environment was built using the Terra/Google Cloud platform, so users familiar with this system may experience shorter onboarding periods. Data exploration and analysis are supported through the use of Jupyter notebooks[32] and RStudio,[33] which are commonly used tools in the field of data analytics and statistical analysis. AnVIL also supports genomics tools, such as Galaxy,[34] for users with less experience in programming who are interested in genomic analysis and provides access to standard command line tools like GATK[35] to facilitate advanced data processing.

Although the potential benefits of using a platform like the AnVIL for sequence data storage, sharing, and analysis are numerous, the unfamiliarity of the platform may limit the ability of investigators to anticipate exactly how data might be shared and/or used and may therefore make early-stage decisions about data modeling and sharing difficult. For example, the automatic linkage of survey, phenotypic, and sequence data in a shared cloud workspace is a novel concept, and investigators will undoubtedly need to make challenging decisions regarding the best way(s) to prepare, share, and utilize such data. Large clinical genomics research consortia like the eMERGE Network and the Implementing Genomics in Practice (IGNITE) Consortium will likely face similar challenges to those experienced by CSER, and the AnVIL platform will be a valuable space for investigators from all disciplines to unite and support one another in this new generation of genomic data sharing and analytics (lesson learned 5b).

Discussion

Recommendations

After dedicating much time and effort to developing and implementing strategies for harmonizing and coordinating consortium-wide datasets, the CSER consortium is well positioned to contribute an impactful and wide-reaching dataset to facilitate research in medical genomics. While the DCC developed tailored strategies to facilitate CSER data coordination, the principles behind these strategies are applicable to other research settings in which data are pooled from heterogeneous sources. Table 2 lists 11 overarching needs and recommendations for conducting multi-site data coordination at the levels of planning, communication, informatics, and data analytics. The following section explores these recommendations through the lens of four thematic domains that emerged from this work: (1) transparency and translation; (2) team morale, collaboration, and trust building; (3) iterative design; and (4) data governance. We also offer guidance on how these recommendations might generalize to projects of different sizes with diverse data coordination needs and capabilities.

Transparency and translation

Clear and consistent communication on the part of research leadership and data coordination teams should be a high priority, from project conception to completion. Ideally, funding opportunity announcements (FOAs) issued by funding agencies should plan for and communicate data-sharing expectations (planning, recommendation 1) to allow research sites to budget and plan for data coordination activities (planning, recommendation 2). When possible, the DCC should be involved in the research planning phase and should continually facilitate conversations surrounding data collection, QA/QC, reporting, modeling, and sharing, so that research sites are sufficiently prepared to participate in data sharing at all project stages (planning, recommendation 3). Given the availability of appropriate experience and expertise, the DCC may act as a stakeholder proxy across research sites and working groups and facilitate data coordination conversations and decision-making. As a liaison between project stakeholders, the CSER DCC was ideally positioned to assume the role of "translator" and facilitate adaptive communication between groups with unique roles and areas of expertise (communication, recommendation 6). Translation should also take place between the consortium and the greater scientific community, since data in controlled-access repositories are expected to have a lifespan beyond the consortium from which they originate. As such, clear documentation of shared data and resources should be developed to encourage appropriate data use and alert users to any unusual or unique data elements prior to analysis (analytics, recommendation 10).

The translator also has a responsibility to communicate data needs centrally and concisely. Separate lines of communication that request different (but related) data coordination action items should be avoided, and requests should instead be aggregated and contextualized with one another (communication, recommendation 4). The expected contributions of stakeholders to different data

Table 2. Recommendations for consortium data coordination

Category	Needs	Recommendations
Planning	clear expectations for internal and external data sharing	1. Build data sharing expectations into expected scope of work in funding announcements (NIH[a])
	sufficient financial resources and time for data coordination	2. Budget for data coordination, management, and reporting at individual research sites (NIH[a])
	integration between DCC and consortium	3. Establish DCC at start of funding period, if not before (NIH[a] and DCC[a])
Communication	consolidation of communication channels	4. Consolidate lines of communication from DCC to working groups and assign action items appropriately (DCC[a] and sites[a])
	technical specifications for data sharing	5. Maximize transparency of data coordination expectations and resources (NIH[a] and DCC[a])
	efficient use of diverse expertise available within the consortium	6. Facilitate translation of critical information between stakeholder groups (DCC[a])
Informatics	consolidation of informatics platforms for data coordination	7. Deploy a secure, centralized web resource for data coordination (DCC[a])
	flexibility in response to unforeseen events and changing analysis plans	8. Build flexibility into central databases and data-management software (DCC[a])
	correct implementation of site-level security and privacy agreements	9. Prioritize data privacy and security during platform design (DCC[a])
Analytics	high-quality and reliable data from heterogeneous sources	10. Provide clear and detailed documentation of shared data resources (DCC[a] and sites[a])
	integration of research and clinical practice; enhanced protection of data from vulnerable populations	11. Document approaches to data governance (DCC[a] and sites[a])

[a]Entities which should be responsible for each recommendation.

coordination activities should also be transparent, both to increase task accountability and to assess the equitable distribution of tasks across the consortium (communication, recommendation 5). Stakeholder communication should be a two-way, responsive process in which DCC processes are adjusted in response to stakeholder feedback and vice versa.

Team morale, collaboration, and trust building

An often-overlooked aspect of data coordination is the importance of interpersonal relationships and team morale within and between stakeholder groups. Making expectations transparent and achievable is critical to demonstrating respect and appreciation for team members' time and efforts (communication, recommendation 5). Similarly, giving team members the space and time to regularly voice ideas and concerns to the leadership and data coordination team is essential for maintaining a culture of mutual respect and understanding across stakeholder groups. Decisions that will impact research workflows and workloads of consortium members should be made mutually and transparently, both to demonstrate respect for one another's time and to avoid situations in which stakeholders must retrospectively address issues introduced earlier in the research process due to a lack of communication or collaborative planning. Strengthening these interpersonal relationships is essential for building a culture of trust within the research team and facilitating a positive data-sharing experience.

Iterative design

Access points to important data coordination tools and resources should be consolidated to minimize burden placed on sites and improve resource transparency (informatics, recommendation 7). Each resource should also be designed to withstand frequent modifications, both on the database and user-interface ends, to accommodate inevitable changes in consortium needs (informatics, recommendation 8). Building iterative design principles into the platform-development process is far more effective at achieving a useful and usable system than deploying a static, pre-designed system.[36] Based on the Gould and Lewis principles of design,[37] system development should involve (1) early focus on endpoint users, (2) early deployment and usability testing, and (3) iterative system design. Employing these principles in practice will help end users identify critical features and potential issues on a rolling basis and ensure that the resulting data coordination system is designed appropriately for the intended user base. However, platform security should remain the highest priority throughout the design process, and design decisions should never be made at the expense of security features (informatics, recommendation 9).

Data governance

While there is an understanding among scientific communities worldwide that sharing research data is a necessary component of scientific progress, the mechanisms for protecting against potential harm while maximizing

usefulness are not well-defined.[38] These two aims are often in tension and lend themselves to diverse data governance strategies across research projects within and between scientific disciplines. In genomics research studies, data-governance frameworks that promote scientific progress should (1) enable data access, (2) follow national laws and international agreements, (3) support appropriate data use, (4) promote equity in the access and analysis of data, and (5) use data for public benefit.[39] However, when operationalizing data-governance frameworks within research consortia, major tensions exist in the areas of data access control, de-identification, and consent models. Combined with the technical challenges of cleaning, harmonizing, and annotating datasets, these tensions contribute to a disconnect between the intent to share data and real-life data-sharing practices.[40] While it is tempting to trace this disconnect to a lack of clear guidance from national agencies and project funders, guidelines like those found in the NIH GDS policy are left intentionally vague to account for vast contextual differences between research projects. To develop a reusable set of data-governance guidelines that can accommodate different research settings and contexts, it may therefore be useful for research projects to document their own approaches to the five components of effective data-governance frameworks listed above and for funding agencies to then develop comprehensive guidelines that accommodate the unique data-governance requirements of diverse research settings (analytics, recommendation 11).

One important tension that arises in clinical research is the need to accommodate varying data-governance expectations across clinical and research settings, particularly for participant privacy and informed consent for data sharing. For example, the Federal Policy for the Protection of Human Subjects (also known as the "Common Rule") is a set of federal regulations that dictates requirements for the ethical management and distribution of data collected from human research subjects, while the HIPAA Privacy Rule is a federal law that enforces standards for the protection of patient medical data. While these regulations are intended to complement one another in clinical research settings, the details of how each set of rules should be applied to the operational components of a data-governance strategy are not well defined, leading to potential gaps in data protections.[41] The US Department of Health and Human Services itself recognizes that "institutions, IRBs and investigators are frequently faced with applying both the Common Rule and the HIPAA Privacy Rule" when making decisions about clinical research protocols, since there are currently no formalized guidelines for merging these requirements.[42] The inclusion of genome and exome sequencing in clinical research further complicates questions of subject and biospecimen identifiability, for which guidance from the Common Rule and HIPAA is limited.[43,44]

In the case of informed consent for data sharing, the details and implications of policies that govern data protections should be made transparent to clinical research participants who are asked to consent to broad data sharing, but researchers and policymakers themselves are still grappling with these details. For example, on the FAQ page of the NIH GDS policy description, a common perception among genomic researchers is that the "NIH requires that investigators obtain consent for broad data sharing and that the participant is disqualified from participating in the study if consent is not obtained," although the NIH clarifies on the same page that this was not the intent of the policy.[45] In addition to questions of appropriate data sharing, the appropriate breadth and depth of information communicated during the informed consent process is challenging to pinpoint, given that it is extremely difficult—if not impossible—to predict exactly how genomic information will be used by researchers in the future. There is an even greater urgency for clarity in genomic data sharing consent procedures for patient populations that are historically marginalized and disadvantaged by biomedical research and medical practice.[46] For example, there is concern among US Indigenous communities that participating in genomic research and sharing genomic data may lead to inappropriate use of that data in the future, leading to imbalanced societal benefits or even harm to those communities.[47] Data governance frameworks that support paradigms like data sovereignty for marginalized populations and dynamic consent procedures may help mitigate some of the risks posed by evolving consent details in medical genomics research.[48]

Generalizability of recommendations

While these recommendations were designed to generalize to other multi-site research projects, we recognize that smaller or less well-funded projects may not be able—or even need—to implement all of the recommendations. For example, a smaller project with two homogeneous research sites (e.g., similar participant populations, research aims, and institutional policies) may not need to establish a formal DCC (recommendation 3) or deploy a multi-user web application (recommendations 7–9). However, the same project would still benefit from having a dedicated group of investigators to oversee data coordination, encourage communication, and facilitate documentation (recommendations 4–6, 10, and 11). While the costs of these recommendations pale in comparison to funding an entire DCC or developing a web application, they are nontrivial. A "bare bones" implementation of a data coordination core would require part-time participation of at least one investigator at each site with data science expertise (similar to the CSER Data Wranglers), one investigator at each site with detailed knowledge about the study (similar to the CSER Project Managers), and one central coordinator to facilitate communication and track progress. As funding agencies increasingly expect research projects to contribute high-quality, harmonized data to public repositories, funders and researchers alike should recognize these dedicated groups as an essential component of any research program and provide

appropriate budget support accordingly (recommendations 1 and 2).

Research projects should consider how the size, complexity, and privacy considerations of their anticipated datasets impact the relative importance of different data coordination needs (see the "needs" column in Table 2) and implement recommendations accordingly. While dataset factors are partly influenced by the number of sites involved in a project, they are not defined by project size. For example, a project with two sites collecting 100 data types (variables, file types, etc.) might have a greater need for more robust data coordination tools than a project with 100 sites collecting two data types. Similarly, smaller consortia collecting data on a large number of participants at each site may have more complex needs than larger consortia collecting data on a small number of participants. However, as the CSER consortium experienced, data coordination needs evolve as the project evolves. Projects should periodically re-evaluate how well their current approaches are addressing their needs and seek additional funding and/or personnel to help implement more rigorous coordination approaches as needed.

Finally, while these recommendations are most translatable to NIH-funded projects within the United States, the basic principles still apply to non-NIH-funded and multi-national projects. Other types of projects may have data-sharing expectations and policies that differ considerably from those of NIH-funded projects, but using well-reasoned communication and informatics practices is ubiquitously beneficial for managing heterogeneous datasets. For example, a 2017 report by the Organisation for Economic Co-operation and Development identified common challenges across 32 international research data networks, including the need for clear roles and responsibilities, transparency, mutual respect, and clear data-governance plans.[49] However, multi-national consortia like the Global Enteric Multicenter Study (GEMS) and the International Cancer Genome Consortium (ICGC) have cited additional challenges—like navigating differences in language, culture, and data-transfer policies between countries—that the current recommendations do not address.[50,51] While privately funded projects may not be required to share data as a condition of funding, they will likely receive requests from peer-reviewed journals to share data before publishing. In this way, the evolving culture of data transparency within the scientific community itself necessitates data coordination.

Conclusions

Data coordination is key to harnessing the full potential of multi-site research projects, yet there exist few guidelines for how the coordination process should be executed. The CSER Data Coordinating Center faced a host of challenges while aggregating common measures data and genomic sequence data across clinical projects and developed a suite of communication and informatics techniques to address these challenges. CSER is not alone in its data coordination odyssey. Other collaborative research projects face similarly complex decision points, and the CSER experience provides insight into how those complexities may be addressed or even prevented with early action.

Data and code availability

The datasets generated during this study are available on the AnVIL cloud platform and can be explored using the AnVIL Dataset Catalog at https://anvilproject.org/data. There are restrictions to the availability of CSER data because they contain demographic, clinical, and raw sequencing information. Access to CSER data is therefore moderated through dbGaP data access requests. To learn more about requesting access to controlled-access AnVIL datasets through dbGaP, please visit https://anvilproject.org/learn/accessing-data/requesting-data-access. The following phsIDs are associated with CSER consortium data: phs002111.v1.p1 (CHARM), phs001089.v3.p1 (SouthSeq), phs002324.v1.p1 (P^3EGS), phs002337.v1.p1 (NYCKidSeq), phs002110.v1.p1 (NCGENES 2), phs002378.v1.p1 (KidsCanSeq), and phs000971.v3.p1 (ClinSeq). The code supporting the current study has not been deposited in a public repository because it is under active development but is available from the corresponding author upon request.

Supplemental information

Supplemental information can be found online at https://doi.org/10.1016/j.xhgg.2022.100120.

Acknowledgments

CSER is supported by the following grants: U01 HG007292, U01 HG007301, U01 HG009599, U01 HG009610, U01 HG006487, U01 HG006485, and U24 HG007307. ITHS is supported by the National Center for Advancing Translational Sciences of the National Institutes of Health under award number UL1 TR002319.

Declaration of interests

The authors declare no competing interests.

Received: March 18, 2022
Accepted: May 16, 2022

References

1. ACMG Board of Directors (2017). Laboratory and clinical genomic data sharing is crucial to improving genetic health care: a position statement of the American College of Medical Genetics and Genomics. Genet. Med. *19*, 721–722. https://doi.org/10.1038/gim.2016.196.
2. Bozeman, B., Fay, D., and Slade, C.P. (2013). Research collaboration in universities and academic entrepreneurship: the-state-of-the-art. J. Technol. Transf. *38*, 1–67. https://doi.org/10.1007/s10961-012-9281-8.
3. Boland, M.R., Karczewski, K.J., and Tatonetti, N.P. (2017). Ten simple rules to enable multi-site collaborations through data sharing. PLoS Comput. Biol. *13*, e1005278. https://doi.org/10.1371/journal.pcbi.1005278.

4. Genomic Data Sharing Policy Team (2014). NIH Genomic Data Sharing Policy (NIH Grants). https://grants.nih.gov/grants/guide/notice-Files/not-Od-14-124.html.
5. Oliver, J.M., Slashinski, M.J., Wang, T., Kelly, P.A., Hilsenbeck, S.G., and McGuire, A.L. (2012). Balancing the risks and benefits of genomic data sharing: genome research participants' perspectives. Public Health Genomics *15*, 106–114. https://doi.org/10.1159/000334718.
6. Byrd, J.B., Greene, A.C., Prasad, D.V., Jiang, X., and Greene, C.S. (2020). Responsible, practical genomic data sharing that accelerates research. Nat. Rev. Genet. *21*, 615–629. https://doi.org/10.1038/s41576-020-0257-5.
7. eMERGE Consortium (2021). Lessons learned from the eMERGE Network: balancing genomics in discovery and practice. Human Genetics Genomics Adv. *2*, 100018. https://doi.org/10.1016/j.xhgg.2020.100018.
8. Cronin-Stubbs, D., DeKosky, S.T., Morris, J.C., and Evans, D.A. (2000). Promoting interactions with basic scientists and clinicians: the NIA Alzheimer's disease data coordinating center. Stat. Med. *19*, 1453–1461. https://doi.org/10.1002/(sici)1097-0258(20000615/30)19:11/12<1453::aid-sim437>3.0.co;2-7.
9. Williford, W.O., Collins, J.F., Horney, A., Kirk, G., McSherry, F., Spence, E., Stinnett, S., Howell, C.L., Garg, R., Egan, D., et al. (2003). The role of the data coordinating center in the DIG trial. Clin. Trials *24*, 277S–288S. https://doi.org/10.1016/s0197-2456(03)00103-x.
10. Washington, N.L., Stinson, E.O., Perry, M.D., Ruzanov, P., Contrino, S., Smith, R., Zha, Z., Lyne, R., Carr, A., Lloyd, P., et al. (2011). The modENCODE Data Coordination Center: lessons in harvesting comprehensive experimental details. Database *2011*, bar023. https://doi.org/10.1093/database/bar023.
11. Mai, P.L., Sand, S.R., Saha, N., Oberti, M., Dolafi, T., DiGianni, L., Root, E.J., Kong, X., Bremer, R.C., Santiago, K.M., et al. (2020). Li-fraumeni exploration consortium data coordinating center: building an interactive web-based resource for collaborative international cancer epidemiology research for a rare condition. Cancer Epidemiol. Biomark. Prev. *29*, 927–935. https://doi.org/10.1158/1055-9965.epi-19-1113.
12. Bonomi, L., Huang, Y., and Ohno-Machado, L. (2020). Privacy challenges and research opportunities for genomic data sharing. Nat. Genet. *52*, 646–654. https://doi.org/10.1038/s41588-020-0651-0.
13. Amendola, L.M., Berg, J.S., Horowitz, C.R., Angelo, F., Bensen, J.T., Biesecker, B.B., Biesecker, L.G., Cooper, G.M., East, K., Filipski, K., et al. (2018). The clinical sequencing evidence-generating research consortium: integrating genomic sequencing in diverse and medically underserved populations. Am. J. Hum. Genet. *103*, 319–327. https://doi.org/10.1016/j.ajhg.2018.08.007.
14. Goddard, K.A.B., Angelo, F.A.N., Ackerman, S.L., Berg, J.S., Biesecker, B.B., Danila, M.I., East, K.M., Hindorff, L.A., Horowitz, C.R., Hunter, J.E., et al. (2020). Lessons learned about harmonizing survey measures for the CSER consortium. J. Clin. Transl. Sci. *4*, 537–546. https://doi.org/10.1017/cts.2020.41.
15. Clinical Sequence Evidence-Generating Research Consortium (2018). CSER Research Materials (CSER). https://cser-consortium.org/cser-research-materials.
16. Harris, P.A., Taylor, R., Thielke, R., Payne, J., Gonzalez, N., and Conde, J.G. (2009). Research electronic data capture (REDCap)–a metadata-driven methodology and workflow process for providing translational research informatics support. J. Biomed. Inform. *42*, 377–381. https://doi.org/10.1016/j.jbi.2008.08.010.
17. Harris, P.A., Taylor, R., Minor, B.L., Elliott, V., Fernandez, M., O'Neal, L., McLeod, L., Delacqua, G., Delacqua, F., Kirby, J., et al. (2019). The REDCap consortium: building an international community of software platform partners. J. Biomed. Inform. *95*, 103208. https://doi.org/10.1016/j.jbi.2019.103208.
18. Chang, W., Cheng, J., Allaire, J.J., Sievert, C., Schloerke, B., Xie, Y., Allen, J., McPherson, J., Dipert, A., and Borges, B. (2021). Web Application Framework for R [R Package Shiny version 1.6.0].
19. Schatz, M.C., Philippakis, A.A., Afgan, E., Banks, E., Carey, V.J., Carroll, R.J., Culotti, A., Ellrott, K., Goecks, J., Grossman, R.L., et al. (2022). Inverting the model of genomics data sharing with the NHGRI genomic data science analysis, visualization, and informatics lab-space. Cell Genomics *2*, 100085. https://doi.org/10.1016/j.xgen.2021.100085.
20. National Human Genome Research Institute (NHGRI) (2019). Notice of New NIH-Designated Data Repository: NHGRI's Genomic Data Science Analysis, Visualization, and Informatics Lab-Space (AnVIL). NIH Grants. https://grants.nih.gov/grants/guide/notice-files/NOT-HG-19-024.html.
21. Global Alliance for Genomics and Health (2016). GENOMICS. A federated ecosystem for sharing genomic, clinical data. Science *352*, 1278–1280. https://doi.org/10.1126/science.aaf6162.
22. NIH Office of Science Policy (2021). NIH GDS Policy Oversight. NIH Office of Science Policy. https://osp.od.nih.gov/scientific-sharing/policy-oversight/.
23. Terra Team (2021). Terra Home Page. Terra. https://app.terra.bio.
24. Nutter, B., and Lane, S. (2018). redcapAPI: Accessing Data from REDCap Projects Using the API (CRAN). https://cran.r-project.org/web/packages/redcapAPI/index.html.
25. Office for Civil Rights (OCR). (2008). Summary of the HIPAA Privacy Rule (HHS.gov). https://www.hhs.gov/hipaa/for-Professionals/privacy/laws-Regulations/index.html.
26. dbGaP Team (2022). dbGaP study submission guide (NCBI). https://www.ncbi.nlm.nih.gov/gap/docs/submissionguide/.
27. NIH Office of Science Policy (2015). Points to Consider in Developing Effective Data Use Limitation Statements (NIH Office of Science Policy). https://sharing.nih.gov/sites/default/files/flmngr/NIH_PTC_in_Developing_DUL_Statements.pdf.
28. Dyke, S.O.M., Philippakis, A.A., Rambla De Argila, J., Paltoo, D.N., Luetkemeier, E.S., Knoppers, B.M., Brookes, A.J., Spalding, J.D., Thompson, M., Roos, M., et al. (2016). Consent codes: upholding standard data use conditions. PLoS Genet. *12*, e1005772. https://doi.org/10.1371/journal.pgen.1005772.
29. Langmead, B., and Nellore, A. (2018). Cloud computing for genomic data analysis and collaboration. Nat. Rev. Genet. *19*, 208–219. https://doi.org/10.1038/nrg.2017.113.
30. National Human Genome Research Institute (NHGRI) (2015). Notice for Use of Cloud Computing Services for Storage and Analysis of Controlled-Access Data Subject to the NIH Genomic Data Sharing (GDS) Policy (NIH Grants). https://grants.nih.gov/grants/guide/notice-Files/not-Od-15-086.html.
31. Carter, A.B. (2019). Considerations for genomic data privacy and security when working in the cloud. J. Mol. Diagn. *21*, 542–552. https://doi.org/10.1016/j.jmoldx.2018.07.009.

32. Kluyver, T., Ragan-Kelley, B., Pérez, F., Granger, B., Bussonnier, M., Frederic, J., Kelley, K., Hamrick, J., Grout, J., Corlay, S., et al. (2016). Jupyter Notebooks - a publishing format for reproducible computational workflows. In Positioning and Power in Academic Publishing: Players, Agents and Agendas, F. Loizides and B. Scmidt, eds. (IOS Press), pp. 87–90.
33. RStudio Team (2021). RStudio: Integrated Development Environment for R (RStudio, PBC).
34. Afgan, E., Baker, D., Batut, B., van den Beek, M., Bouvier, D., Cech, M., Chilton, J., Clements, D., Coraor, N., Grüning, B.A., et al. (2018). The Galaxy platform for accessible, reproducible and collaborative biomedical analyses: 2018 update. Nucleic Acids Res. *46*, W537–W544. https://doi.org/10.1093/nar/gky379.
35. Van der Auwera, G.A., and O'Connor, B.D. (2020). Genomics in the Cloud: Using Docker, GATK, and WDL in Terra (O'Reilly Media).
36. Nielsen, J. (1993). Iterative user-interface design. Computer *26*, 32–41. https://doi.org/10.1109/2.241424.
37. Gould, J.D., and Lewis, C. (1985). Designing for usability: key principles and what designers think. Commun. ACM *28*, 300–311. https://doi.org/10.1145/3166.3170.
38. Tenopir, C., Rice, N.M., Allard, S., Baird, L., Borycz, J., Christian, L., Grant, B., Olendorf, R., and Sandusky, R.J. (2020). Data sharing, management, use, and reuse: practices and perceptions of scientists worldwide. PLoS One *15*, e0229003. https://doi.org/10.1371/journal.pone.0229003.
39. O'Doherty, K.C., Shabani, M., Dove, E.S., Bentzen, H.B., Borry, P., Burgess, M.M., Chalmers, D., De Vries, J., Eckstein, L., Fullerton, S.M., et al. (2021). Toward better governance of human genomic data. Nat. Genet. *53*, 2–8. https://doi.org/10.1038/s41588-020-00742-6.
40. Danchev, V., Min, Y., Borghi, J., Baiocchi, M., and Ioannidis, J.P.A. (2021). Evaluation of data sharing after implementation of the international committee of medical journal editors data sharing statement requirement. JAMA Netw. Open *4*, e2033972. https://doi.org/10.1001/jamanetworkopen.2020.33972.
41. Wolf, L.E., Hammack, C.M., Brown, E.F., Brelsford, K.M., and Beskow, L.M. (2020). Protecting participants in genomic research: understanding the "web of protections" afforded by federal and state law. J. Law Med. Ethics *48*, 126–141. https://doi.org/10.1177/1073110520917000.
42. US Department of Health & Human Services Office for Human Research Protections; and The Secretary's Advisory Committee on Human Research Protections (SACHRP) (2011). Attachment D: FAQ's Terms and Recommendations on Informed Consent and Research Use of Biospecimens (HHS.gov). https://www.hhs.gov/ohrp/sachrp-committee/recommendations/2011-October-13-Letter-Attachment-D/index.html.
43. Evans, B.J., and Jarvik, G.P. (2018). Impact of HIPAA's minimum necessary standard on genomic data sharing. Genet. Med. *20*, 531–535. https://doi.org/10.1038/gim.2017.141.
44. Fisher, C.B., and Layman, D.M. (2018). Genomics, big data, and broad consent: a new ethics frontier for prevention science. Prev. Sci. *19*, 871–879. https://doi.org/10.1007/s11121-018-0944-z.
45. NIH Office of Science Policy (2015). Genomic Data Sharing Policy FAQs: Consent for Broad Sharing (OSP Website). https://sharing.nih.gov/faqs#/genomic-data-sharing-policy.htm.
46. Norstad, M., Outram, S., Brown, J.E.H., Zamora, A.N., Koenig, B.A., Risch, N., Norton, M.E., Slavotinek, A., and Ackerman, S.L. (2022). The difficulties of broad data sharing in genomic medicine: empirical evidence from diverse participants in prenatal and pediatric clinical genomics research. Genet. Med. *24*, 410–418. https://doi.org/10.1016/j.gim.2021.09.021.
47. Fox, K. (2020). The illusion of inclusion - the "all of us" research program and indigenous peoples' DNA. N. Engl. J. Med. *383*, 411–413. https://doi.org/10.1056/nejmp1915987.
48. Kaye, J., Whitley, E.A., Lund, D., Morrison, M., Teare, H., and Melham, K. (2015). Dynamic consent: a patient interface for twenty-first century research networks. Eur. J. Hum. Genet. *23*, 141–146. https://doi.org/10.1038/ejhg.2014.71.
49. OECD (2017). Co-ordination and Support of International Research Data Networks (Organisation for Economic Co-Operation and Development (OECD)). https://one.oecd.org/document/DSTI/STP/GSF(2017)5/en/pdf.
50. Biswas, K., Carty, C., Horney, R., Nasrin, D., Farag, T.H., Kotloff, K.L., and Levine, M.M. (2012). Data management and other logistical challenges for the GEMS: the data coordinating center perspective. Clin. Infect. Dis. *55*, S254–S261. https://doi.org/10.1093/cid/cis755.
51. Phillips, M., Molnár-Gábor, F., Korbel, J.O., Thorogood, A., Joly, Y., Chalmers, D., Townend, D., and Knoppers, B.M. (2020). Genomics: data sharing needs an international code of conduct. Nature *578*, 31–33. https://doi.org/10.1038/d41586-020-00082-9.

HGGA, Volume *3*

Supplemental information

Lessons learned and recommendations for data coordination in collaborative research: The CSER consortium experience

Kathleen D. Muenzen, Laura M. Amendola, Tia L. Kauffman, Kathleen F. Mittendorf, Jeannette T. Bensen, Flavia Chen, Richard Green, Bradford C. Powell, Mark Kvale, Frank Angelo, Laura Farnan, Stephanie M. Fullerton, Jill O. Robinson, Tianran Li, Priyanka Murali, James M.J. Lawlor, Jeffrey Ou, Lucia A. Hindorff, Gail P. Jarvik, and David R. Crosslin

Figure S1. CSER projects, site populations and sequencing modalities.

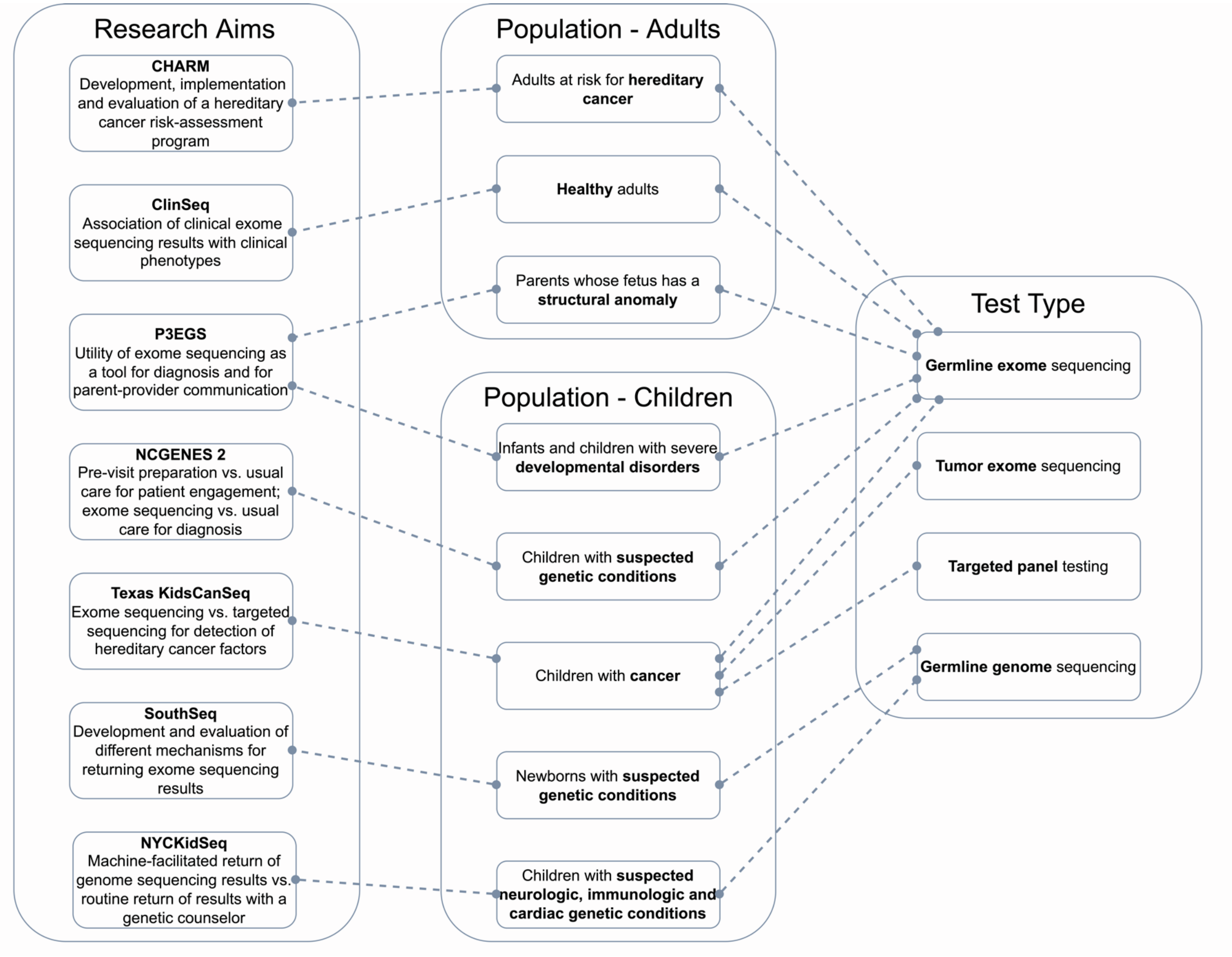

Figure S2. Survey administration timepoints for CSER harmonized survey measures.

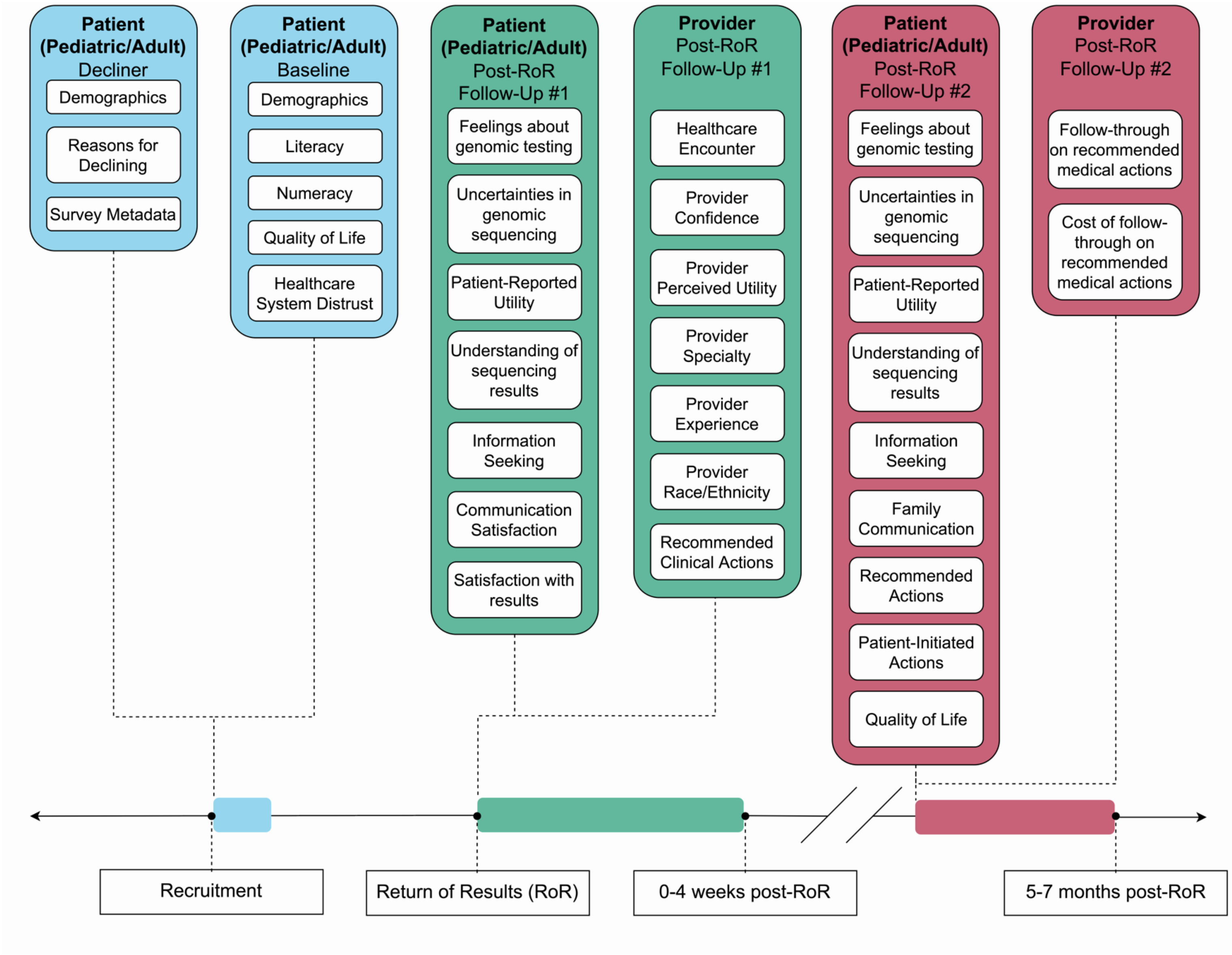

Figure S3. Reporting timepoints for genomic sequencing results, both at the participant level and at the case level.

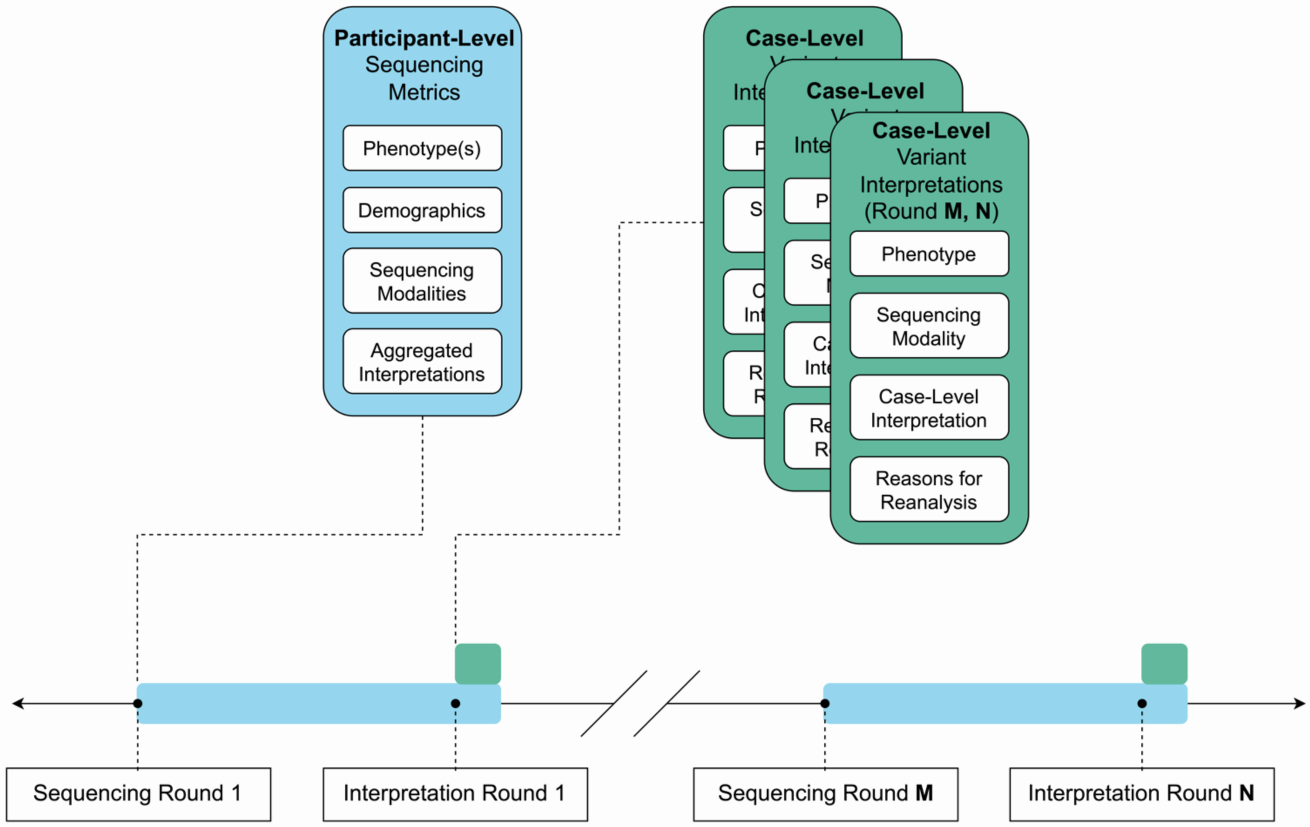

Figure S4. Timeline of the harmonized measure change proposal process and implementation of the post-Return of Results (RoR) to follow-up survey elapsed time variables.

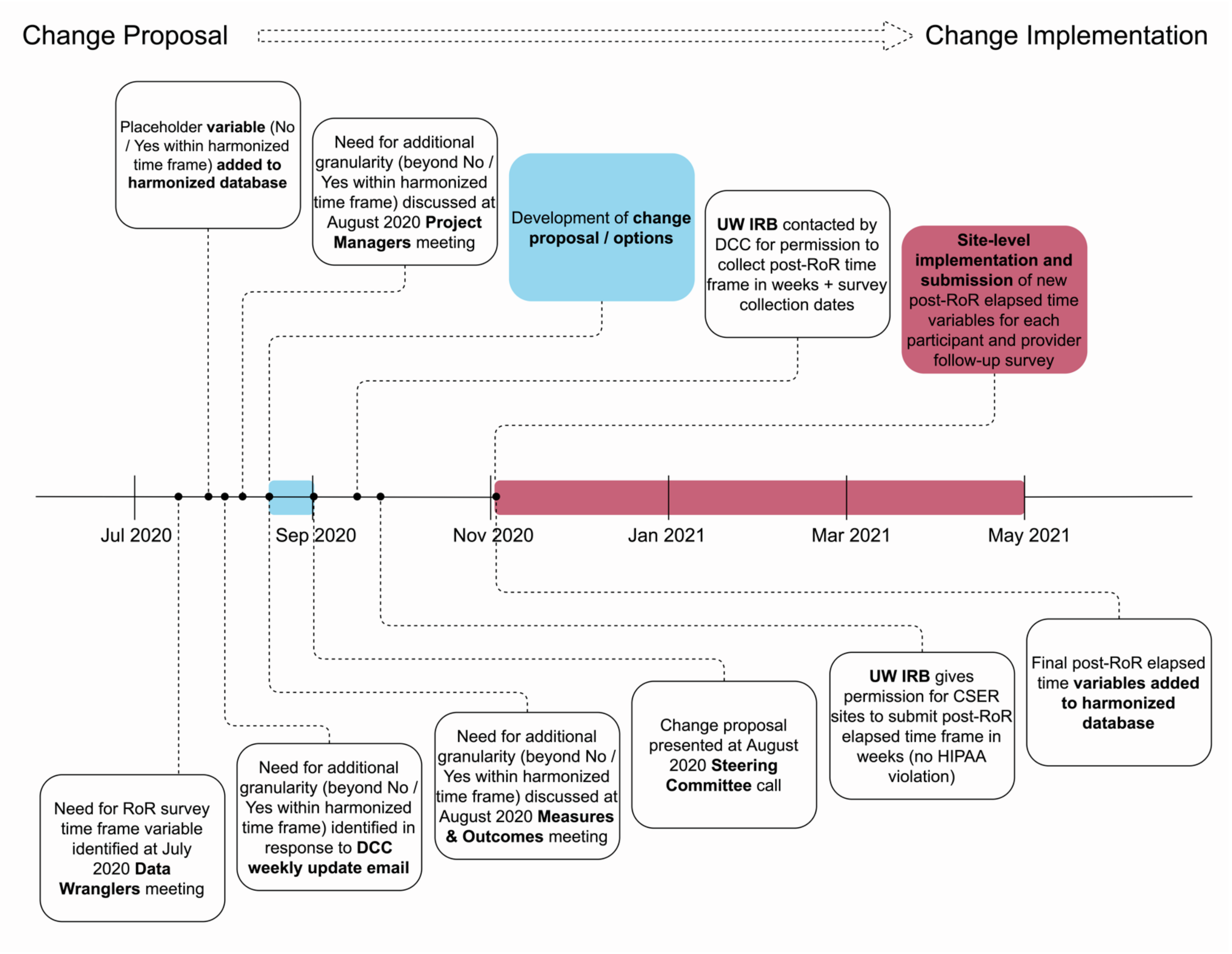

Figure S5. Data upload interface on the CSER Data Hub website.

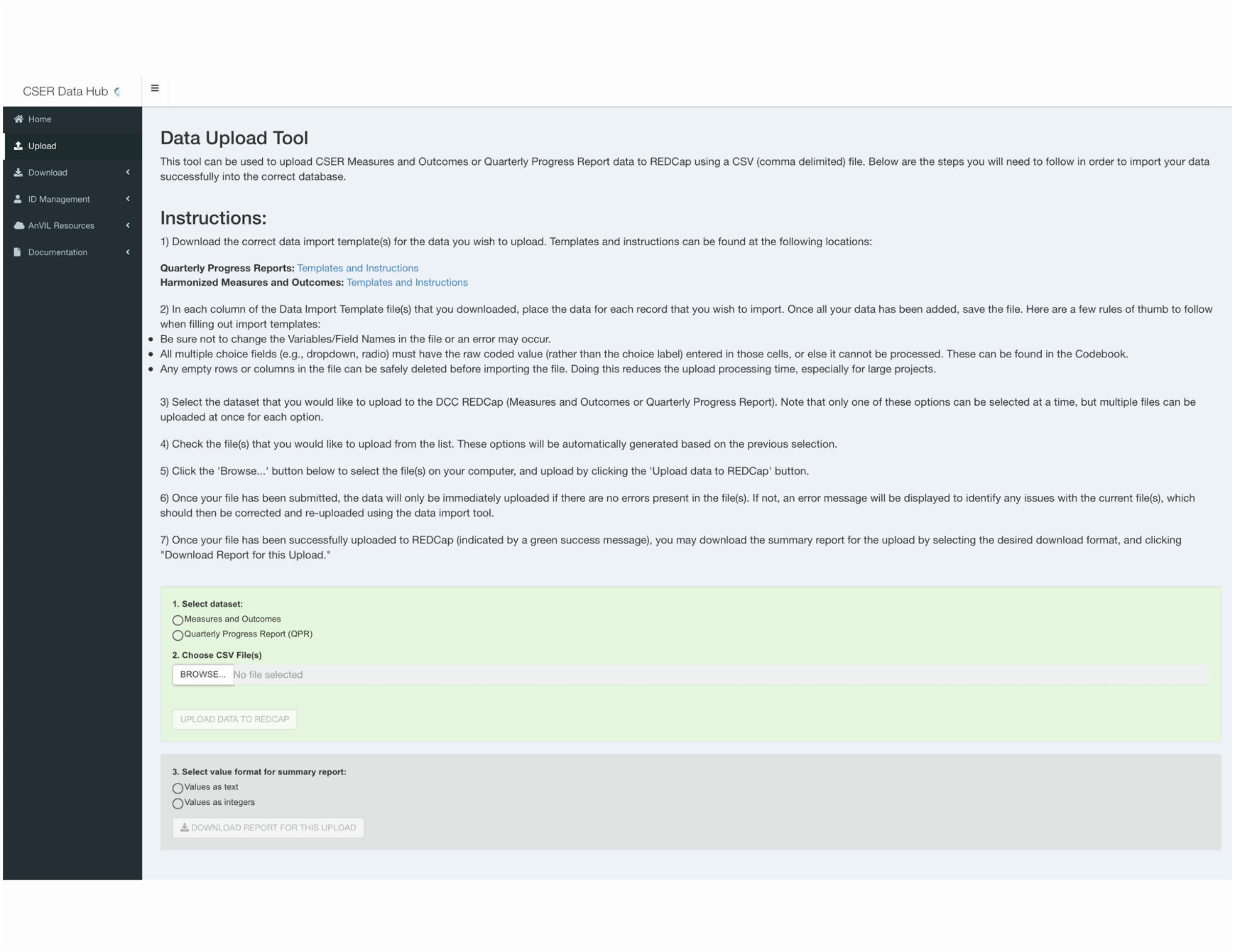

Figure S6. Multi-site harmonized data download interface on the CSER Data Hub website.

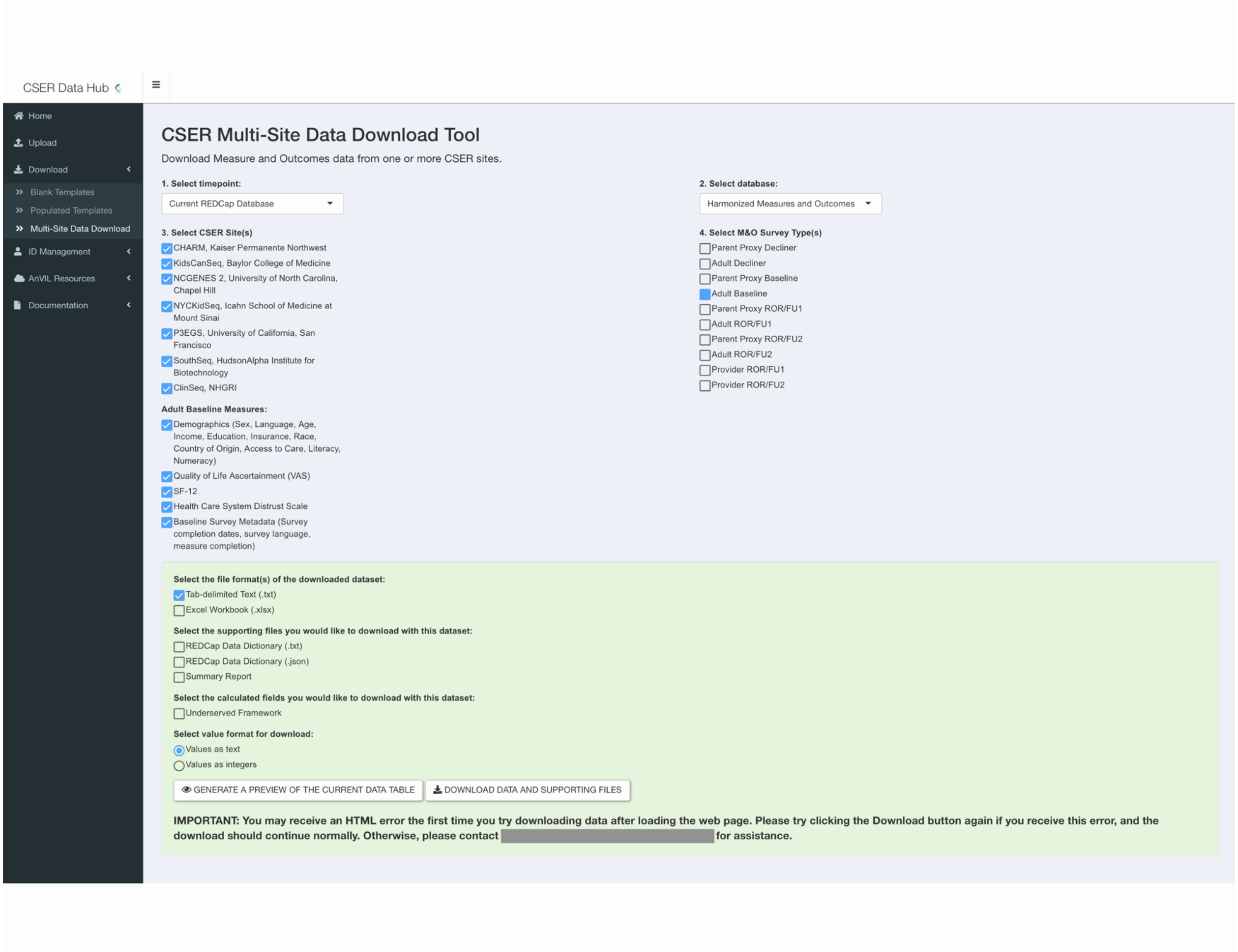

Figure S7. CSER ID management interface on the CSER Data Hub website.

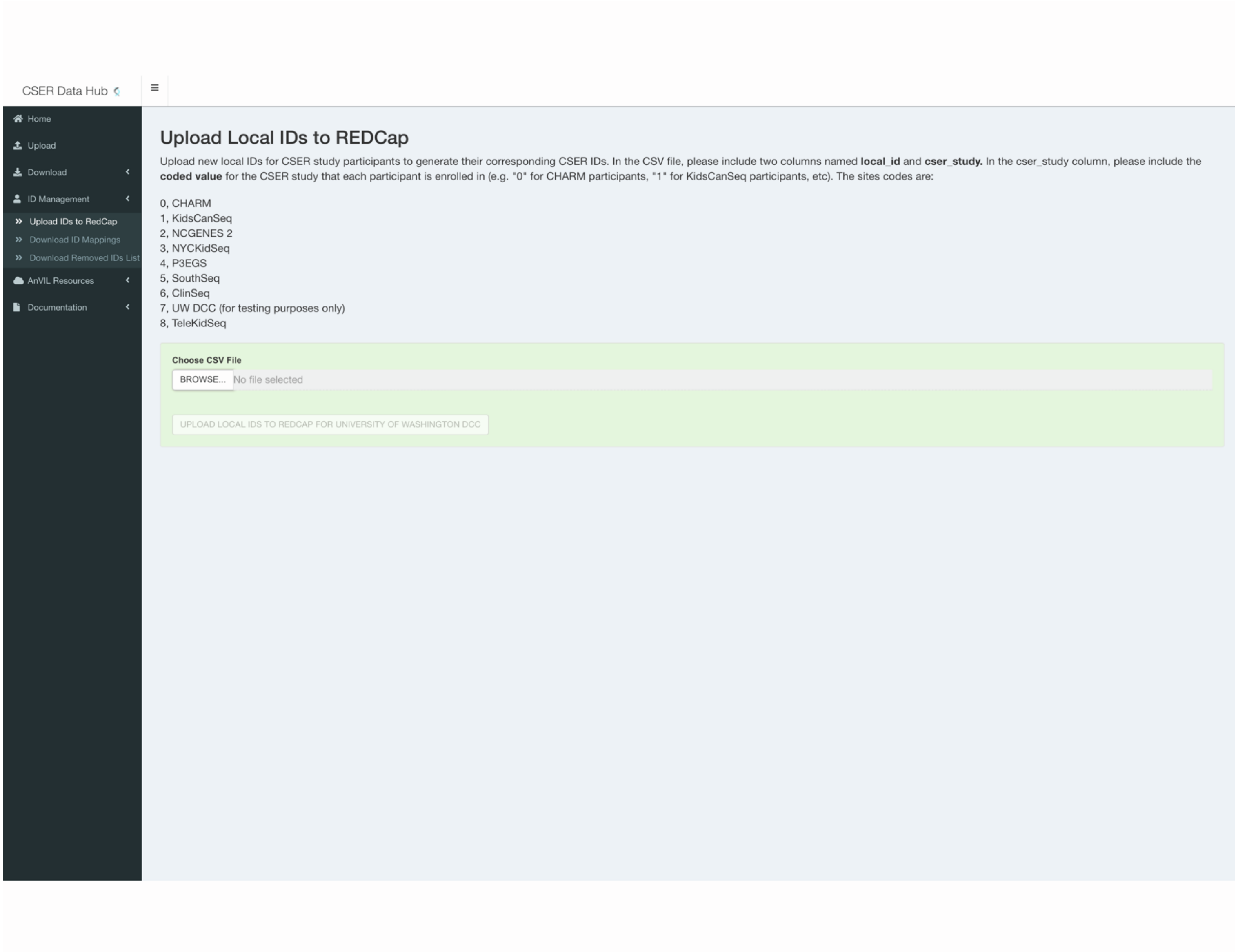

Figure S8. Sequence data upload instructions on the CSER Data Hub website.

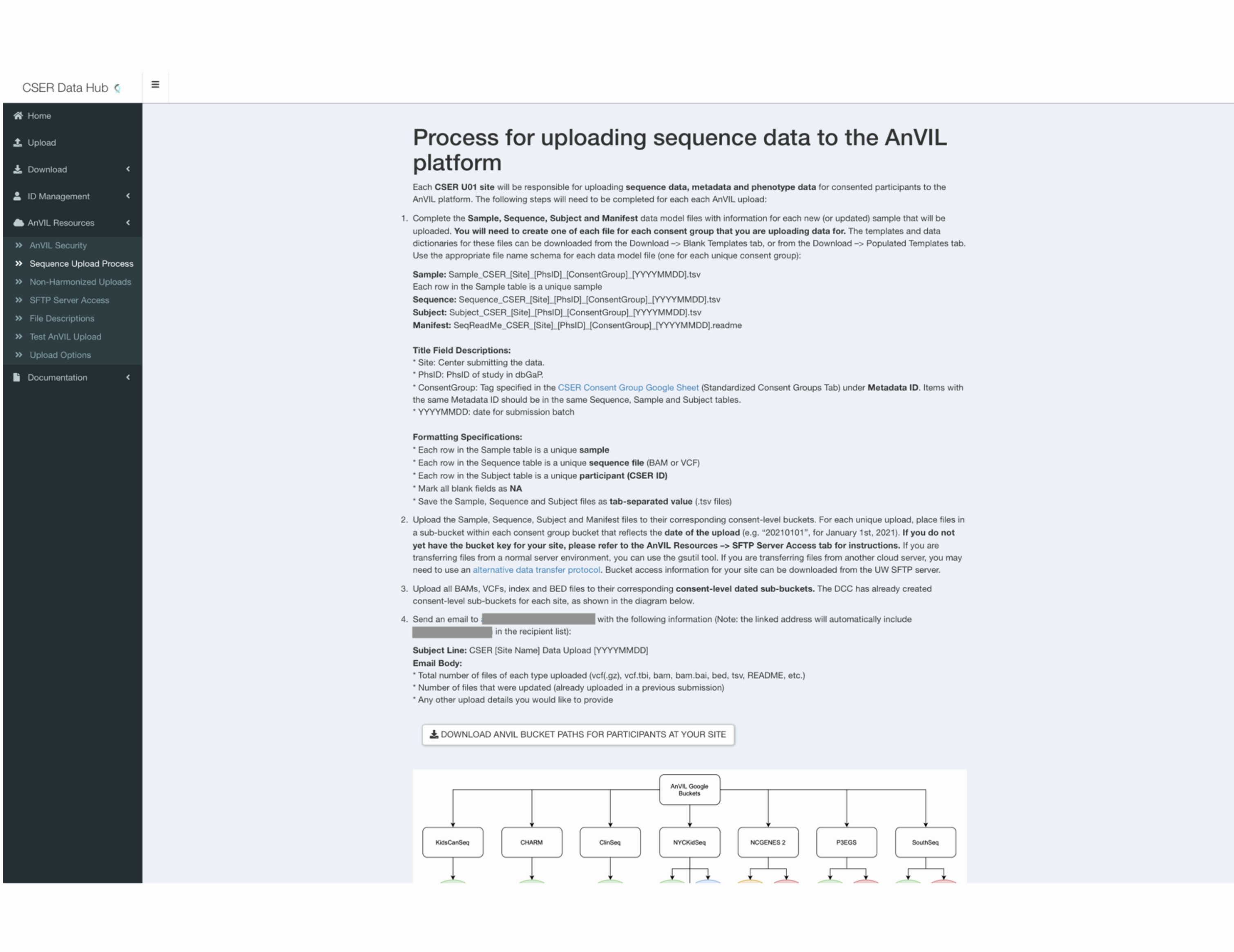

CSER Data Hub

- Home
- Upload
- Download
- ID Management
- AnVIL Resources
  - AnVIL Security
  - Sequence Upload Process
  - Non-Harmonized Uploads
  - SFTP Server Access
  - File Descriptions
  - Test AnVIL Upload
  - Upload Options
- Documentation

Process for uploading sequence data to the AnVIL platform

Each **CSER U01 site** will be responsible for uploading **sequence data, metadata and phenotype data** for consented participants to the AnVIL platform. The following steps will need to be completed for each each AnVIL upload:

1. Complete the **Sample, Sequence, Subject and Manifest** data model files with information for each new (or updated) sample that will be uploaded. **You will need to create one of each file for each consent group that you are uploading data for.** The templates and data dictionaries for these files can be downloaded from the Download –> Blank Templates tab, or from the Download –> Populated Templates tab. Use the appropriate file name schema for each data model file (one for each unique consent group):

   Sample: Sample_CSER_[Site]_[PhsID]_[ConsentGroup]_[YYYYMMDD].tsv
   Each row in the Sample table is a unique sample
   Sequence: Sequence_CSER_[Site]_[PhsID]_[ConsentGroup]_[YYYYMMDD].tsv
   Subject: Subject_CSER_[Site]_[PhsID]_[ConsentGroup]_[YYYYMMDD].tsv
   Manifest: SeqReadMe_CSER_[Site]_[PhsID]_[ConsentGroup]_[YYYYMMDD].readme

   Title Field Descriptions:
   * Site: Center submitting the data.
   * PhsID: PhsID of study in dbGaP.
   * ConsentGroup: Tag specified in the CSER Consent Group Google Sheet (Standardized Consent Groups Tab) under **Metadata ID**. Items with the same Metadata ID should be in the same Sequence, Sample and Subject tables.
   * YYYYMMDD: date for submission batch

   Formatting Specifications:
   * Each row in the Sample table is a unique **sample**
   * Each row in the Sequence table is a unique **sequence file** (BAM or VCF)
   * Each row in the Subject table is a unique **participant (CSER ID)**
   * Mark all blank fields as **NA**
   * Save the Sample, Sequence and Subject files as **tab-separated value** (.tsv files)
2. Upload the Sample, Sequence, Subject and Manifest files to their corresponding consent-level buckets. For each unique upload, place files in a sub-bucket within each consent group bucket that reflects the **date of the upload** (e.g. "20210101", for January 1st, 2021). **If you do not yet have the bucket key for your site, please refer to the AnVIL Resources –> SFTP Server Access tab for instructions.** If you are transferring files from a normal server environment, you can use the gsutil tool. If you are transferring files from another cloud server, you may need to use an alternative data transfer protocol. Bucket access information for your site can be downloaded from the UW SFTP server.
3. Upload all BAMs, VCFs, index and BED files to their corresponding **consent-level dated sub-buckets.** The DCC has already created consent-level sub-buckets for each site, as shown in the diagram below.
4. Send an email to [redacted] with the following information (Note: the linked address will automatically include [redacted] in the recipient list):

   Subject Line: CSER [Site Name] Data Upload [YYYYMMDD]
   Email Body:
   * Total number of files of each type uploaded (vcf(.gz), vcf.tbi, bam, bam.bai, bed, tsv, README, etc.)
   * Number of files that were updated (already uploaded in a previous submission)
   * Any other upload details you would like to provide

DOWNLOAD ANVIL BUCKET PATHS FOR PARTICIPANTS AT YOUR SITE

Figure S9. Change log documentation on the CSER Data Hub website.

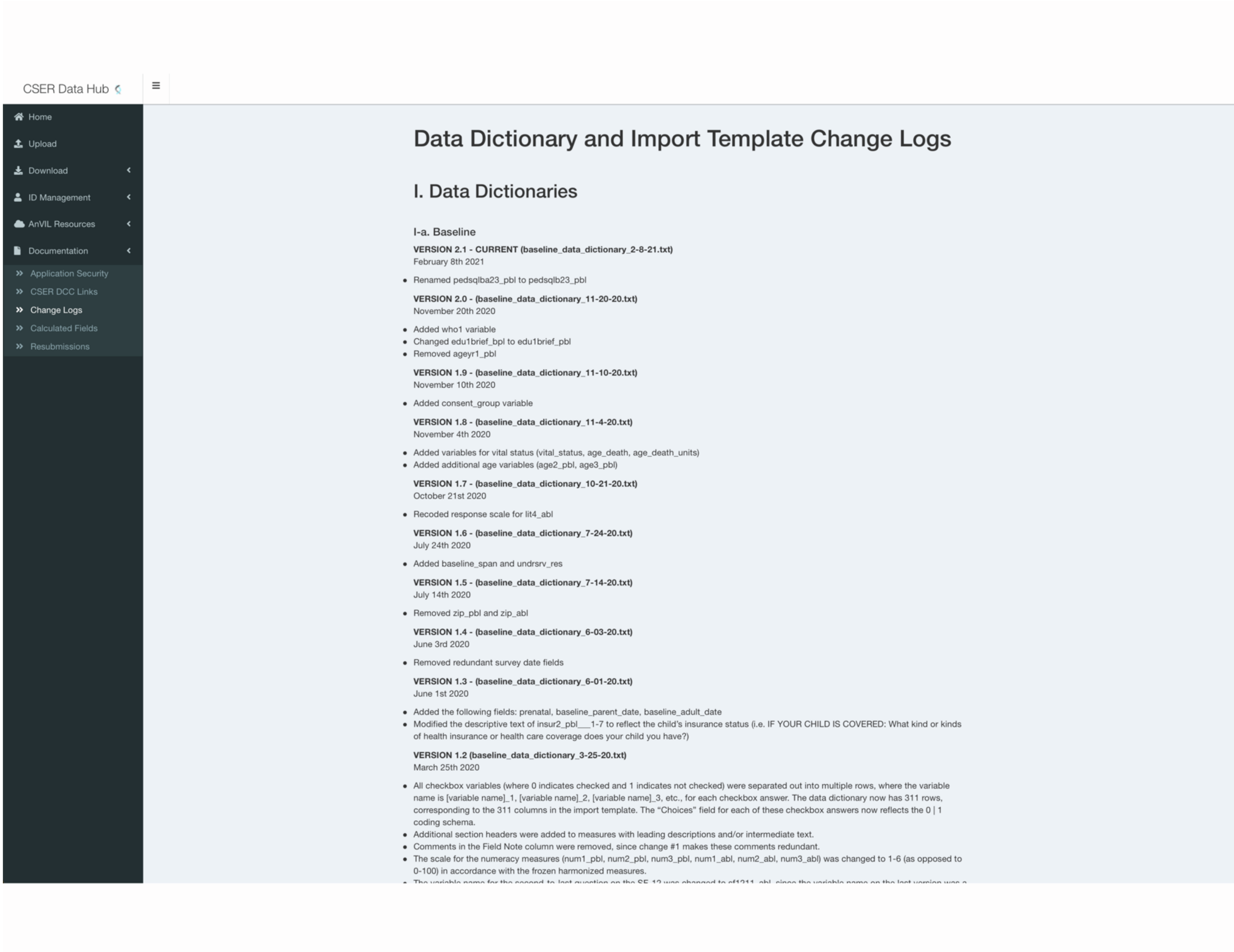

CSER Data Hub

Home
Upload
Download
ID Management
AnVIL Resources
Documentation
Application Security
CSER DCC Links
Change Logs
Calculated Fields
Resubmissions

Data Dictionary and Import Template Change Logs

I. Data Dictionaries

I-a. Baseline

VERSION 2.1 - CURRENT (baseline_data_dictionary_2-8-21.txt)
February 8th 2021

- Renamed pedsqlba23_pbl to pedsqlb23_pbl

VERSION 2.0 - (baseline_data_dictionary_11-20-20.txt)
November 20th 2020

- Added who1 variable
- Changed edu1brief_bpl to edu1brief_pbl
- Removed ageyr1_pbl

VERSION 1.9 - (baseline_data_dictionary_11-10-20.txt)
November 10th 2020

- Added consent_group variable

VERSION 1.8 - (baseline_data_dictionary_11-4-20.txt)
November 4th 2020

- Added variables for vital status (vital_status, age_death, age_death_units)
- Added additional age variables (age2_pbl, age3_pbl)

VERSION 1.7 - (baseline_data_dictionary_10-21-20.txt)
October 21st 2020

- Recoded response scale for lit4_abl

VERSION 1.6 - (baseline_data_dictionary_7-24-20.txt)
July 24th 2020

- Added baseline_span and undrsrv_res

VERSION 1.5 - (baseline_data_dictionary_7-14-20.txt)
July 14th 2020

- Removed zip_pbl and zip_abl

VERSION 1.4 - (baseline_data_dictionary_6-03-20.txt)
June 3rd 2020

- Removed redundant survey date fields

VERSION 1.3 - (baseline_data_dictionary_6-01-20.txt)
June 1st 2020

- Added the following fields: prenatal, baseline_parent_date, baseline_adult_date
- Modified the descriptive text of insur2_pbl___1-7 to reflect the child's insurance status (i.e. IF YOUR CHILD IS COVERED: What kind or kinds of health insurance or health care coverage does your child you have?)

VERSION 1.2 (baseline_data_dictionary_3-25-20.txt)
March 25th 2020

- All checkbox variables (where 0 indicates checked and 1 indicates not checked) were separated out into multiple rows, where the variable name is [variable name]_1, [variable name]_2, [variable name]_3, etc., for each checkbox answer. The data dictionary now has 311 rows, corresponding to the 311 columns in the import template. The "Choices" field for each of these checkbox answers now reflects the 0 | 1 coding schema.
- Additional section headers were added to measures with leading descriptions and/or intermediate text.
- Comments in the Field Note column were removed, since change #1 makes these comments redundant.
- The scale for the numeracy measures (num1_pbl, num2_pbl, num3_pbl, num1_abl, num2_abl, num3_abl) was changed to 1-6 (as opposed to 0-100) in accordance with the frozen harmonized measures.
- [illegible]

Figure S10. Reference sheet for Baseline Measures in the CSER cross-site Adaptation Dictionary.

BASELINE						
	CHARM	Baylor	UCSF	HUDSONALPHA	Sinai/NYCKidSeq	NCGENES/UNC
Gender	A	A	A	D	SA	A
**~~DOB~~**						
Age year					SA	
Language	A	A	A	?	SA	A
Income	B	B			SA	
Education	B	B	B		SA	
Insurance	SA	SA	SA	SA	SA	
Country of origin	B	D?		D	A	D
Access	A			D	SA	
Literacy		A			SA	
Numeracy			D		SA	
Race/Ethnicity			D		SA	
R/E parent 1	NA		A			
R/E/parent 2	NA	D	A			D
**~~Zip~~**						
VAS			A	A		SA
SF-12		NA	NA	NA	NA	NA
PEDSQL	NA	D	D	D	SA	

**Legend**		**Notes**
Identical to harmonized item and response scale =		No changes to harmonized items or response scale
Identical to harmonized item and response scale. Brief version used =	B	Collapsed/Brief harmonized version used for this item
Slight Adaptation=	SA	Slight change in question, question format or response scale. Ex: changed formatting, slight changes to wording of question or response scale
Adaptation=	A	Significant change in question, question format or response scale. Ex: dropped or changed items, changed responses
Dropped=	D	Whole survey dropped
Removed from data base=		Items were removed due to changes in privacy policy
Scale not applicable to study population=		Ex: Adult scale in pediatric population

Table S1. Examples of modifications, additions, and transformations to the harmonized CSER survey measures and outcomes database.

See attached Excel spreadsheet.

Table S2. Harmonized sequence and sample metadata model.

See attached Excel spreadsheet.

Table S3. CSER harmonized consent groups. DUC = dbGaP Data Use Category; DUR = Data Use Restriction; GRU = General Research Use; HMB = Health/medical/biomedical research; IRB = Ethics Approval Required.

	**Survey Data**		**Sequence Data**	
**Consent Group**	**DUC**	**DUR**	**DUC**	**DUR**
1	GRU	None	GRU	None
2	GRU	IRB	GRU	IRB
3	GRU	IRB	N/A	CSER-ONLY Access
4	GRU	IRB	N/A	N/A
5	N/A	CSER-ONLY Access	GRU	IRB
6	N/A	CSER-ONLY Access	N/A	CSER-ONLY Access
7	N/A	CSER-ONLY Access	N/A	N/A
8	HMB	None	HMB	None
